# Amplifiable protein identification via residue-resolved barcoding and composition code counting

**DOI:** 10.1093/nsr/nwae183

**Published:** 2024-05-28

**Authors:** Weiming Guo, Yuan Liu, Yu Han, Huan Tang, Xinyuan Fan, Chu Wang, Peng R Chen

**Affiliations:** Synthetic and Functional Biomolecules Center, Beijing National Laboratory for Molecular Sciences, Key Laboratory of Bioorganic Chemistry and Molecular Engineering of Ministry of Education, College of Chemistry and Molecular Engineering, Peking University, Beijing 100871, China; Synthetic and Functional Biomolecules Center, Beijing National Laboratory for Molecular Sciences, Key Laboratory of Bioorganic Chemistry and Molecular Engineering of Ministry of Education, College of Chemistry and Molecular Engineering, Peking University, Beijing 100871, China; Synthetic and Functional Biomolecules Center, Beijing National Laboratory for Molecular Sciences, Key Laboratory of Bioorganic Chemistry and Molecular Engineering of Ministry of Education, College of Chemistry and Molecular Engineering, Peking University, Beijing 100871, China; Synthetic and Functional Biomolecules Center, Beijing National Laboratory for Molecular Sciences, Key Laboratory of Bioorganic Chemistry and Molecular Engineering of Ministry of Education, College of Chemistry and Molecular Engineering, Peking University, Beijing 100871, China; Synthetic and Functional Biomolecules Center, Beijing National Laboratory for Molecular Sciences, Key Laboratory of Bioorganic Chemistry and Molecular Engineering of Ministry of Education, College of Chemistry and Molecular Engineering, Peking University, Beijing 100871, China; Synthetic and Functional Biomolecules Center, Beijing National Laboratory for Molecular Sciences, Key Laboratory of Bioorganic Chemistry and Molecular Engineering of Ministry of Education, College of Chemistry and Molecular Engineering, Peking University, Beijing 100871, China; Peking-Tsinghua Center for Life Sciences, Academy for Advanced Interdisciplinary Studies, Peking University, Beijing 100871, China; Synthetic and Functional Biomolecules Center, Beijing National Laboratory for Molecular Sciences, Key Laboratory of Bioorganic Chemistry and Molecular Engineering of Ministry of Education, College of Chemistry and Molecular Engineering, Peking University, Beijing 100871, China; Peking-Tsinghua Center for Life Sciences, Academy for Advanced Interdisciplinary Studies, Peking University, Beijing 100871, China

**Keywords:** protein identification, amplifiable fingerprinting, residue-specific chemistry, DNA barcoding, composition code counting

## Abstract

Ultrasensitive protein identification is of paramount importance in basic research and clinical diagnostics but remains extremely challenging. A key bottleneck in preventing single-molecule protein sequencing is that, unlike the revolutionary nucleic acid sequencing methods that rely on the polymerase chain reaction (PCR) to amplify DNA and RNA molecules, protein molecules cannot be directly amplified. Decoding the proteins via amplification of certain fingerprints rather than the intact protein sequence thus represents an appealing alternative choice to address this formidable challenge. Herein, we report a proof-of-concept method that relies on residue-resolved DNA barcoding and composition code counting for amplifiable protein fingerprinting (AmproCode). In AmproCode, selective types of residues on peptides or proteins are chemically labeled with a DNA barcode, which can be amplified and quantified via quantitative PCR. The operation generates a relative ratio as the residue-resolved ‘composition code’ for each target protein that can be utilized as the fingerprint to determine its identity from the proteome database. We developed a database searching algorithm and applied it to assess the coverage of the whole proteome and secretome via computational simulations, proving the theoretical feasibility of AmproCode. We then designed the residue-specific DNA barcoding and amplification workflow, and identified different synthetic model peptides found in the secretome at as low as the fmol/L level for demonstration. These results build the foundation for an unprecedented amplifiable protein fingerprinting method. We believe that, in the future, AmproCode could ultimately realize single-molecule amplifiable identification of trace complex samples without further purification, and it may open a new avenue in the development of next-generation protein sequencing techniques.

## Teaser

AmproCode transfers non-amplifiable proteins into amplifiable molecules to magnify their amino-acid-composition signals for ultrasensitive protein identification, which might open a new avenue in the development of next-generation protein sequencing techniques.

## INTRODUCTION

Ultrasensitive protein identifications will bring breakthrough technology to life sciences as well as propelling clinical diagnostics. Although the rapid development of mass spectrometry-based proteomics strategies has greatly facilitated protein identification from complex samples, low-abundance proteins from trace samples often fail to be identified due to the detection limit and dynamic range of mass spectrometry [1,2]. The similar challenges in genomics and transcriptomics have been successfully overcome by using nucleic acid amplification methods. For example, in the next-generation sequencing, the polymerase chain reaction (PCR) and other amplification methods have allowed quantitative DNA and RNA analysis from low copy numbers [3], which has revolutionized genomics and transcriptomics research [4]. However, we are still waiting for a similar breakthrough in protein amplification and identification.

Since traditional protein identification methods such as mass spectrometry cannot fill the gap, researchers are making efforts to develop alternative novel protein analysis methods [5,6]. The emerging methods, such as single-molecule Edman degradation [7,8], N-terminal amino-acid-specific binding probes [9], nanopore analysis [10–17], recognition tunneling [18,19], single-molecule mass spectrometry [20,21], DNA-nanotechnology-based protein identification and so on [22–25], can be classified as single-molecule protein identification [26,27]. These methods have great potential in ultrasensitive protein identification, but all of them rely on the ultrasensitive single-molecule analytical instruments to detect signals from a single amino acid, peptide or protein.

We envision that an alternative solution to the problem could be amplifiable protein identification. Although there has been no reported natural machinery to directly replicate proteins from a template, converting the protein sequence information into amplifiable barcodes such as DNA might be an approach. Previous ‘immuno-PCR’ methods have enabled protein detection by using DNA-antibody conjugates with high specificity (by the antibody) and sensitivity (by DNA amplification) [28,29], but only specific target proteins with available antibodies can be detected. With the upsurge in collaborations between organic chemists and chemical biologists in recent years, many residue-specific reactions have been invented that have broadened the toolbox for precise protein engineering, functional modulation and activity-based proteomics [30–34]. Such metal-trigged, photo-trigged, electro-trigged or spontaneous chemical reactions range from electrophilic and nucleophilic substitution to redox reaction [30–34]. Besides the most well-known reactions for lysine (Lys or K) and cysteine (Cys or C), residue-specific chemistry has also been expanded to various types of amino acids including aspartic acid (Asp or D)/glutamic acids (Glu or E), tyrosine (Tyr or Y), arginine (Arg or R), methionine (Met or M), histidine (His or H), tryptophan (Trp or W), serine (Ser or S) and so on [32–38]. Considering that many newly developed protein identification approaches are based on the database-matching of the protein fingerprints including the number of amino acids [8,24–27], we believe that using DNA barcodes to record part of the sequence information can allow protein fingerprinting identification in an amplifiable and universal manner [39].

**Figure 1.**
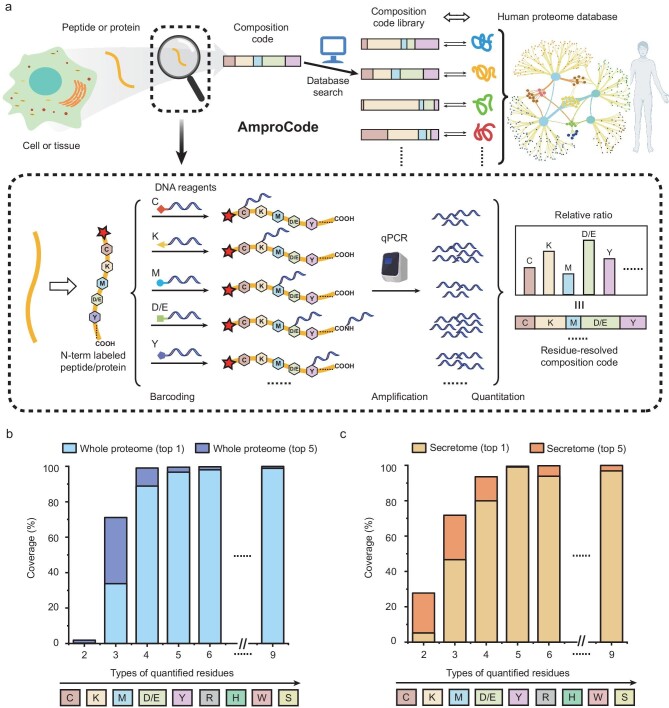
Design and overview of AmproCode. (a) The workflow of amplifiable protein identification through AmproCode. Each type of selected residue on peptide or protein samples is modified by using DNA barcodes, respectively. The residue-resolved composition code is generated by the relative ratio of these residues from each protein, which can be amplified and quantified by using qPCR. The sequence information of all proteins in the human proteome database can be converted into the composition code library. Matching the experimentally obtained composition code with the codes in the database library by using the database search algorithm allows the identification of the sample. (b) Theoretical evaluation of the coverage rate of AmproCode in the whole human proteome. (c) Theoretical evaluation of the coverage rate of AmproCode in the secretome. The residue types in the composition code range from two to nine in the order of Cys, Lys, Met, Asp/Glu, Tyr, Arg, His, Trp and Ser. Targeting the highest single hit and the top five hits in identification are evaluated respectively.

Herein, we report an amplifiable protein fingerprinting method, termed ‘AmproCode’, by integrating residue-resolved DNA barcoding, quantitative DNA amplification, composition code counting and computer-aided database-matching (Fig. 1a). We reasoned that, through residue-specific chemical reactions, several types of residues can be selectively and quantitatively labeled with DNA barcodes, which are leveraged to magnify the fingerprints of trace samples via quantitative PCR (qPCR) amplification. The residue-resolved composition code is generated by the relative ratio of these amino acids in the target protein as measured by using qPCR. Since the sequence information of all proteins in the human proteome database can be converted into a composition code library, AmproCode allows matching between the experimentally obtained composition code and the codes in the library using a customized database search algorithm, which may facilitate amplifiable protein identification.

## RESULTS AND DISCUSSION

### Theoretical justification for AmproCode

We envision that, as proteins vary in their sequences, the relative ratio of their amino acids (the ‘composition code’) can be employed as a unique fingerprint to facilitate protein identification. Inspired by the previous work on protein fingerprinting, we believed that the composition code of a partial set of residues in a given protein would be sufficient for identification. We first established a composition code library from the whole human proteome database (Swiss-Prot) including 20 588 reviewed peptides and proteins with manual annotations [40]. For each protein, its composition code is numerically represented as the ratio of all the nine residue types that can be modified by specific chemical reactions including Cys, Lys, Asp/Glu, Tyr, Arg, Met, His, Trp and Ser [30–38]. We found that >98% of the proteins in the whole human proteome had unique composition codes consisting of these nine residue types, which suggested that nearly all human proteins could be distinguished by their composition codes in theory (Fig. 1b).

We next aimed to establish a proof-of-concept model to evaluate the feasibility of applying AmproCode for protein identification if a partial set of residues are chemically labeled, amplified by DNA barcodes, and accurately quantified in relative composition. For this purpose, we developed a computational tool to compare the input composition code with the code library from the proteome database and ranked the protein entries in the proteome based on the cosine similarity value. Here we used vectors to record the composition codes of the input data and all the protein entries in a proteome database, and the cosine similarity could be calculated by using the following formula:


\begin{eqnarray*}
\textit{cosine}.\textit{similarity} = \frac{{{\boldsymbol{u}} \cdot {\boldsymbol{v}}}}{{\|{\boldsymbol{u}}\| \|{\boldsymbol{v}}\|}}
\end{eqnarray*}

where ***u*** and ***v*** represent two vectors of two composition codes. From the computational analysis, we found that, with an increase in residue types in the composition code, the coverage of AmproCode in the whole proteome improved accordingly. When the residue types increased from three (Cys, Lys and Met) to four (adding Asp/Glu), the theoretical coverage of the human proteome could be raised from 34% to 89% in the absence of experimental errors (Fig. 1b). Besides the composition code of Cys, Lys and Met and Asp/Glu, we also estimated other five combinations and all of them could cover >75% of the human proteins (e.g. 81% for the combination of Cys, Lys and Met and Tyr in Fig. S1). Although, in certain cases, some proteins and peptides may have degenerated composition codes that can compromise the coverage, AmproCode could still narrow down the analyte from the whole proteome to a few candidates that could be further verified. Indeed, if the restrictions on protein identification in this model could be relaxed from targeting the highest single hit (‘top 1’) to the top five hits (‘top 5’) in the code library, then the proteome coverage rate could be raised to 99% using the composition code of Cys, Lys and Met and Asp/Glu (Fig. 1b). Therefore, the composition code containing four residues is sufficient for protein identification.

Often, in practical applications, it is only necessary to analyse a subset of rather than the whole proteome in many biological samples. For example, the secretome is a critical portion of the whole proteome with great clinical relevance, with which many proteins mediate endocrine communication and regulate fundamental homeostatic processes [41], and may serve as a novel biomarker and/or potential drug target for clinical diagnosis and treatment. To investigate the compatibility of AmproCode with the secretome, we also established the composition code library of human secretome including 2675 mature chains or active peptides from 2112 precursor proteins with the annotation of ‘secreted’ in Swiss-Prot. Considering that some secreted peptides are processed from the same protein, we therefore combined them in the same protein entry in the database search. The computational analysis showed that, with the composition code of four residue types, AmproCode also had the capacity for protein identification in the secretome. For example, the composition code of Cys, Lys, Met and Asp/Glu could cover 94% of the secreted proteins (top 5) while the combination of Cys, Lys, Met and Tyr could cover 90% (top 5) (Fig. 1c and Fig. S1). These computational analysis results thus provided the theoretical basis for our method.

### Residue-specific DNA barcoding

Residue-specific chemical reactions laid the ground for AmproCode because DNA barcodes need to be selectively attached to amino acid side chains of the peptide or protein with high specificity and efficiency [42]. Considering the availability, specificity and efficiency of reported residue-specific labeling reactions, we initially tested five residue-specific reactions targeting Cys, Lys, Met, Asp/Glu and Tyr: (i) maleimide reagents to functionalize Cys; (ii) *N*-hydroxysuccinimide (NHS) esters to functionalize Lys; (iii) redox-activated chemical tagging by oxaziridines on Met; (iv) amide condensation on Asp/Glu with the assistance of (7-azabenzotriazol-1-yloxy) tripyrrolidinophosphonium hexafluorophosphate (PyAOP); and (v) formylbenzene diazonium to modify Tyr. These reactions hold certain advantages including the wide availability of regents, high reactivity under mild conditions and good specificity to target residues, and they have been successfully applied in mass spectrometry-based proteomic workflow before [43], suggesting their practicability in AmproCode.

**Figure 2.**
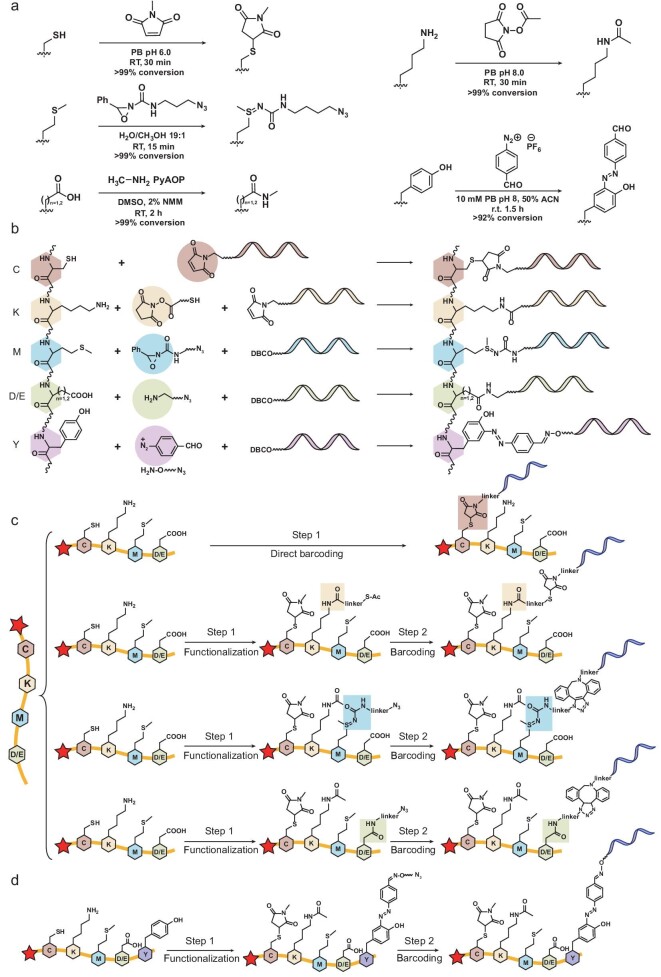
Residue-resolved DNA barcoding on Cys, Lys, Met and Asp/Glu via residue-specific reactions. (a) The scheme and conditions for residue-specific reactions on Cys, Lys, Met, Asp/Glu (as well as C-terminal) and Tyr. (b)–(d) Scheme of residue-specific DNA barcoding on peptides. (b) The DNA barcoding scheme is based on the residue-specific reactions of Cys, Lys, Met, Asp/Glu and Tyr. (c) Cys is directly labeled by methylmaleimide-modified DNA barcodes while Lys, Met and Asp/Glu resides are modified through a two-step, residue-specific functionalization and barcoding procedure, respectively. In the first step, the functional groups including azide and thioester are introduced to the targeted residues while some reactive amino acids are protected. In the second barcoding step, these functional groups are further labeled by using Mal-DNA or DBCO-DNA barcode reagents. (d) The peptide with Tyr residue is modified through a two-step scheme. In the first step, the azide group is introduced to the Tyr residues and, in the second step, the azide group is further labeled by using DBCO-DNA.

In a small-molecule system, each of the five amino acids with the 9-fluorenylmethyloxycarbonyl (Fmoc) group was reacted with the corresponding labeling reagents and the reaction efficiency was measured based on the ultraviolet absorption of the Fmoc group using analytical high-performance liquid chromatography (HPLC). We observed an excellent conversion rate (∼99%) for Cys, Lys, Met and Asp/Glu on the small-molecule level. In comparison, the conversion rate of Tyr was only 92% (Fig. 2a and Figs S2 and S9). Considering that the performance of Tyr was not as good as that of the other four residues and the theoretical justification proved that four residues were sufficient for protein identification, we chose the combination of Cys, Lys, Met and Asp/Glu as the initial combination code and left Tyr as an alternative candidate in AmproCode, especially in certain cases containing the Tyr residue.

We then evaluated the labeling efficiency and selectivity of these reactions on the peptide level. We commercially synthesized a model peptide containing each of the Cys, Lys, Met and Asp/Glu residues. Cys and Lys were modified in a one-pot reaction, followed by Met and Asp modification, respectively. The high efficiency and specificity (>95%) were validated by the ultraviolet absorption using HPLC and mass spectrometry (Figs S3 and S10).

Next, we developed the residue-specific DNA barcoding scheme (Fig. 2b and c, and Fig. S4): (i) Cys residues were directly barcoded by using the maleimide-conjugated DNA reagent (Mal-DNA); (ii) Lys residues were functionalized by using the thioester-modified NHS ester and barcoded by using Mal-DNA in the presence of hydroxylamine (or the azide-modified NHS ester and the dibenzocyclooctyne-conjugated DNA reagent, DBCO-DNA) after protecting Cys by using *N*-methyl maleimide; (iii) Met residues were functionalized by using the azide-modified oxaziridine and barcoded by using DBCO-DNA after protecting Cys and Lys by using *N*-methyl maleimide and the *N*-acetoxysuccinimide, respectively; (iv) Asp/Glu residues as well as the C-termini of the protein were labeled using the azide-modified amine and barcoded by using DBCO-DNA after protecting Cys and Lys. We applied the DNA barcoding strategy on the synthetic model secreted hormone peptide, ELA, which functions in cardiovascular development and homeostasis, and can help reduce maternal mortality [44,45]. The peptide contained all the four types of residues including two Cys residues, two Lys residues, two Met residues and a C-terminal carboxylate. We divided the peptide sample into four aliquots for, respectively, DNA barcoding with the designed common DNA barcode. The products including ELA-C-(DNA)_2_, ELA-K-(DNA)_2_, ELA-M-(DNA)_2_ and ELA-C-terminal-DNA were confirmed by using mass spectrometry (Fig. S11), proving our residue-specific DNA barcoding design.

Furthermore, we also designed the Tyr-specific peptide barcoding workflow based on the azo coupling reaction so that we could have more combination choices to improve proteome coverage and/or facilitate experimental procedures. The Tyr residues were labeled via a one-pot reaction with the formylbenzene diazonium and the azide-modified hydroxylamine reagents, and the azide group attached to residues was further modified by using the DBCO-DNA (Fig. 2b and d). We validated the workflow on a peptide with a Tyr residue (Fig. S5).

### Amplifiable peptide fingerprinting via AmproCode

After accomplishing the residue-specific DNA barcoding, we proceeded to explore amplifiable protein fingerprinting on the ELA peptide. We first tested the qPCR performance of the DNA peptide conjugates including the linear range of detection and bias to each labeled residue (Fig. S6). We found that the linear range of detecting the DNA-barcoded peptide was 10^4^ copies/μL (10 fmol/L) to 10^8^ copies/μL (0.1 nmol/L) by using qPCR with excellent correlation (*R*^2^ > 0.999), suggesting a broad dynamic range (10^5^) and low detection limit (10^4^ copies/μL). We also found that the DNA barcodes that attached to different residues introduced negligible bias to the qPCR.

**Figure 3.**
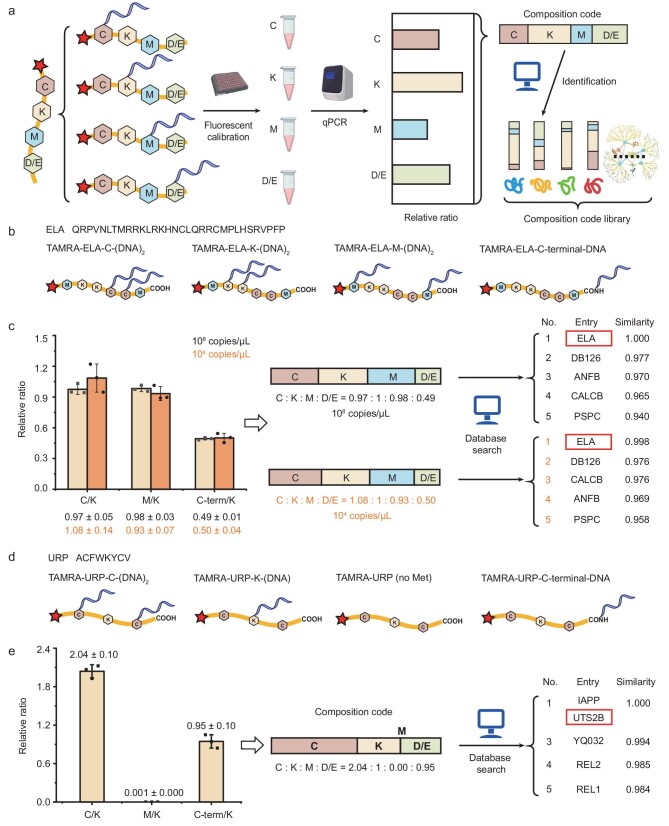
Amplifiable peptide fingerprinting via AmproCode using the composition code of Cys, Lys, Met and Asp/Glu. (a) Schematic illustration of amplifiable peptide fingerprinting through AmproCode. After normalizing the four DNA-barcoded samples using fluorescence, the composition code of Cys, Lys, Met and Asp/Glu of the target protein can be generated by using quantitative PCR amplification, which can be used as the fingerprint to match the composition code library via a database search algorithm. (b) and (c) Identification of the ELA peptide through AmproCode using the composition code of Cys, Lys, Met and Asp/Glu. (b) Four residue types of TAMRA-ELA including Cys, Lys, Met and Asp/Glu/C-terminal are labeled by using the DNA barcode, respectively. (c) qPCR quantified composition code of ELA is 0.97:1:0.98:0.49 at 10^8^ copies/μL (top) and 1.08:1:0.93:0.50 at 10^4^ copies/μL (bottom) (average of three independent replicates). After matching these two composition codes with the code library from the secretome, the ELA peptide (protein entry: ELA) can be identified as the top candidate both at 10^8^ and 10^4^ copies/μL with the highest score. (d) and (e) Identification of the URP peptide through AmproCode using the composition code of Cys, Lys, Met and Asp/Glu. (d) Four residue types of TAMRA-URP including Cys, Lys, Met and Asp/Glu/C-terminal are labeled by using the DNA barcode, respectively. (e) qPCR quantified composition code of URP is 2.04:1:0.00:0.95 at 10^8^ copies/μL (average of three independent replicates). The URP peptide (protein entry: UTS2B) can be identified in the top two candidates with the highest score.

To quantitatively obtain a count ratio of these chemically labeled amino acids based on DNA barcoding signals, we also need to incorporate an internal standard for normalization because, according to the barcoding workflow, the model peptide sample should be divided into four aliquots for DNA barcoding, respectively, and the sample loss after purification and other steps might vary differently. We therefore synthesized a fluorescent dye (e.g. tetramethylrhodamine TAMRA) to the N-terminal of the peptides so that the four residue-specific DNA-barcoded samples could be calibrated by the N-terminal fluorescence (Fig. 3a). Each of the four residue types of the synthetic TAMRA-ELA was chemically labeled with the DNA barcode and amplified by using qPCR, respectively (Fig. 3b and Fig. S12). Through qPCR of samples at 10^8^ copies/μL, the obtained composition code (Cys:Lys:Met:Asp/Glu/C-terminal) of ELA was 0.97:1:0.98:0.49, which was consistent with the theoretical values (1:1:1:0.5). We searched for the obtained code in the library of secretome and found that ELA was identified in the top-five-ranking list with the highest similarity value (Fig. 3c).

After stepwise dilution, we showed that, at a peptide concentration of as low as 10^4^ copies/μL (∼0.07 pg/mL), a similar composition code of the ELA peptide could also be obtained as 1.08:1:0.93:0.50, which was also sufficient to identify the ELA peptide in the secretome (Fig. 3c). Since the detection limit of a common ELISA kit for ELA is ∼1–100 pg/mL and the detection limit of a typical mass spectrometry is 10^6^–10^9^ copies [46,47], the sensitivity of AmproCode is ∼10–10 000 times better than those of the two common protein identification methods despite the fact that we have not yet realized single-molecule amplifiable fingerprinting. These experiments proved that AmproCode had the capability for amplifiable protein identification of a trace sample with extremely low concentrations.

In addition to ELA, we also applied AmproCode to identify another synthetic peptide, URP, which is a potent physiological vasoconstrictor with essential roles in hypertension [48]. After DNA barcoding and qPCR amplification at 10^8^ copies/μL, the obtained composition code (Cys:Lys:Met:Asp/Glu/C-terminal) of URP was 2.04:1:0.00:0.95, which was sufficient to identify URP from the secretome (Fig. 3d and e, and Fig. S13).

### Aβ peptide identification via AmproCode from sample mixtures

**Figure 4.**
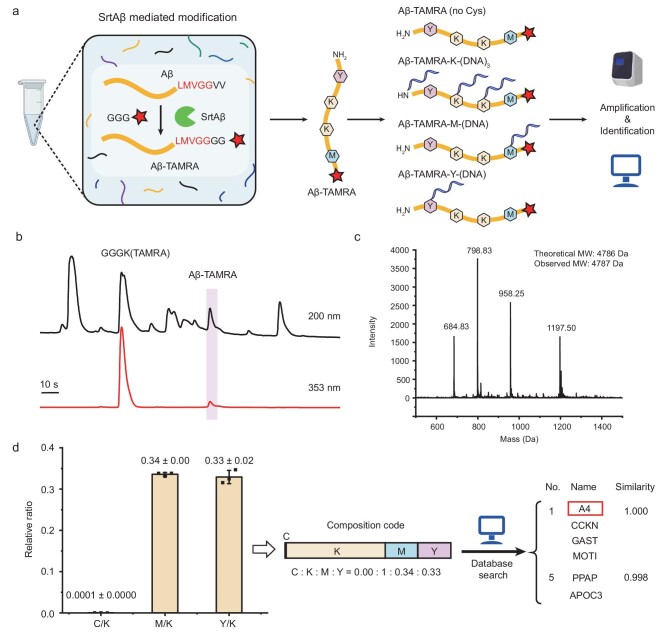
AmproCode for Aβ detection from complex mixtures. (a) Transpeptidase SrtAβ-mediated Aβ modification for AmproCode. In a complex sample, SrtAβ recognizes the LMVGG sequence in the Aβ peptide and conjugates GGG-TAMRA to it. The product Aβ-TAMRA can be applied in the AmproCode workflow. (b) HPLC analysis of the sample mixture after labeling Aβ with TAMRA. The absorption spectra are chosen at 200 and 353 nm (TAMRA-specific absorption). (c) Mass spectrum of Aβ-TAMRA. The theoretical molecule weight (MW) is 4786 Da and the observed MW is 4787 Da. (d) Identification of Aβ peptide via AmproCode using the composition code of Cys, Lys, Met and Tyr. qPCR quantified composition code of Aβ is 0.00:1:0.34:0.33 (Cys:Lys/N-terminal:Met:Y) at 10^8^ copies/μL (average of three independent replicates). After matching this composition code with the code library from the secretome, Aβ (protein entry: A4) can be identified in the top four candidates with the highest score.

We have proved that AmproCode has the capacity to identify purified peptides. In principle, the ultimate version of AmproCode in the future could operate in a single-molecule mode in which a single protein is a pure substance after diluting into an isolated microenvironment such as a microwell on chips (Fig. S8). Moving toward this direction, we attempted to extend the application of the current AmproCode including analysis of some specific target proteins of interest from complex samples after additional isolation or enrichment steps. Aβ peptide was chosen as the detection target because it plays an essential role in Alzheimer's disease (AD) [49] and both soluble Aβ peptide and Aβ plaque are important clinical biomarkers as well as therapeutic targets [50]. More conveniently, SrtAβ, a transpeptidase sortase A variant, has been recently evolved to recognize the LMVGG sequence of the Aβ protein and attach a poly-glycine peptide with functional groups after the LMVGG sequence [51]. We thus aimed to leverage SrtAβ to assist Aβ isolation in a two-step workflow. First, the TAMRA fluorescent dye was conjugated with the Aβ peptide by SrtAβ in a sample mixture, which contained over six protein or peptide components including humanin-like 9, URP, NY-ESO-1 (157–165), Aβ, GGGK(TAMRA), SrtAβ, as well as impurities. Second, the product, Aβ-TAMRA, was then easily isolated from the mixture by using HPLC based on its unique absorption spectrum (Fig. 4a–c and Fig. S7). Moreover, the fluorescence of TAMRA could also be used for sample calibration in the subsequent analysis.

Considering that the Aβ peptide contains six Asp/Glu residues but only one Tyr residue, we reasoned that quantitation of this single Tyr would be more straightforward and accurate than the quantitation of six acidic residues. The Tyr barcoding strategy provided us with the choice to replace Asp/Glu with Tyr to facilitate Aβ identification. After DNA barcoding and qPCR amplification of Cys, Lys, Met and Tyr (Fig. S14), the composition code was quantified as 0.00:1:0.34:0.33, which was consistent with the theoretical ratio of 0:1:0.33:0.33. After the obtained composition code in the database was searched for, Aβ peptide was found among the top-five candidate list (Fig. 4d). Furthermore, as it was reported that SrtAβ had the capacity of modifying Aβ peptide with the biotin affinity handle in human cerebrospinal fluid (CSF) and blood [51], we thought that combining it with SrtAβ for Aβ enrichment and identification might pave the way for AmproCode to be implemented in real-world applications in the future (Fig. S7).

### Simulating the error tolerance of AmproCode

**Figure 5.**
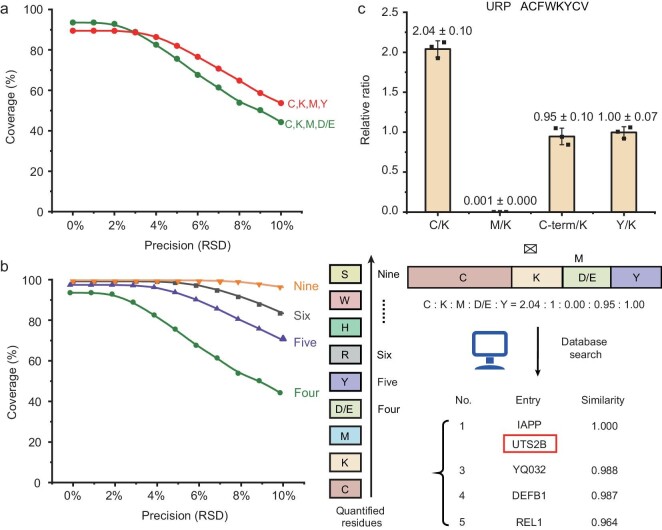
Error tolerance of AmproCode. (a) Simulations of the percentage of correctly identified proteins (coverage) in the secretome via AmproCode using the composition code of Cys, Lys, Met and Asp/Glu (C,K,M,D/E) or Cys, Lys, Met and Tyr (C,K,M,Y) with different experimental precision (0.05%–10% RSD). The coverage represents a statistical average of five independently simulated coverage values. (b) Simulations of the correctly identified protein rates (coverage) of AmproCode in the secretome with different experimental precision (0.05%–10% RSD) and different residue types (four, five, six and nine in the order of Cys, Lys, Met, Asp/Glu, Tyr, Arg, His, Trp and Ser). The coverage represents a statistical average of five independently simulated coverage values. (c) qPCR quantified composition code with Tyr of URP is 2.04:1:0.00:0.95:1.00 at 10^8^ copies/μL (average of three independent replicates). The URP peptide (protein entry: UTS2B) can be identified in the top two candidates with the highest score.

Although we have theoretically analysed the coverage of AmproCode and experimentally identified three model peptides including ELA, URP and Aβ in the secretome database via AmproCode, we also acknowledged that experimental variations that were not considered in our previous computational estimations would influence the protein identification rate to various degrees. Thus, we assessed the secretome coverage of our method that was equal to the corrected identified protein rate (accuracy) by using computational simulations in terms of two parameters: the types of quantitated residues and the experimental precision that was defined as the relative standard deviation (RSD) of the quantitated relative ratio between the selected amino acids in the composition code. We first simulated the coverage using the two sets of composition codes: (i) Cys, Lys, Met, Asp/Glu; and (ii) Cys, Lys, Met, Tyr. Interestingly, although the theoretical coverage of Asp/Glu was higher when the RSD was equal to 0, the composition code with Tyr behaved better than Asp/Glu, with an RSD of ≥3%, indicating that the Tyr residue had a higher error tolerance (Fig. 5a). However, we had to admit that the coverage or the accuracy declined fast with the increasing RSD, even if we chose the Tyr code.

Labeling and quantifying more residues would be an effective way to improve the error tolerance of AmproCode. Based on the computational estimation results, when all the nine residues with specific chemical reactions were labeled and quantified, the coverage could be maintained at >96% even when the RSD was loosened to 10% (Fig. 5b). We found that quantitation of one more residue could bring significant improvement. For example, if Tyr was added to the combination of Cys, Lys, Met and Asp/Glu, then the correct identification rate would rise from 44% to 71% with 10% RSD, representing a 1.6-fold improvement (Fig. 5b), and it could match the accuracy of some other newly reported protein fingerprinting identification methods such as Edman fluorosequencing (40%), DNA nanoswitch calipers (76% with a probability of >90%) and FRET-X (39%–91% under different conditions) [8,24,25]. In one particular case, when we added Tyr to the previously obtained composition code of the URP peptide, the new composition code was generated as 2.04:1:0.00:0.95:1.00 (Cys:Lys:Met:Asp/Glu/C-terminal:Tyr) and the cosine similarity value between the measured composition codes of the URP and the other proteins decreased. For example, the similarity value of the No.3 protein entry ‘YQ032’ decreased from 0.994 (Fig. 3d) to 0.988 (Fig. 5c), suggesting that Tyr quantitation brought greater distinction between other proteins and more error tolerance on protein identification. Since the adaptability of AmproCode is related to the number of residue types, we would like to apply more alternative chemical reactions to modify and quantify more residues (e.g. kethoxal for Arg barcoding) to further increase its coverage and accuracy in the future.

## CONCLUSION

In summary, we have reported a proof-of-concept study of the amplifiable protein fingerprinting method AmproCode, which could magnify the composition code of proteins via residue-resolved DNA barcoding and amplification. Theoretically, we showed that the composition code of several residues on a peptide or protein of interest was adequate for fingerprinting and identification from a database derived from the whole proteome or the secretome. We also estimated the coverage of our method with different experimental parameters by computational simulations, supporting our method under more realistic conditions. Experimentally, we designed the DNA barcode and attached it to several types of amino acids on peptides, including Cys, Lys, Met, Asp/Glu as well as Tyr residues, via a panel of highly efficient and mutually orthogonal residue-specific reactions. The DNA barcode on peptides was amplified and quantified by using qPCR, which yielded the composition code of the analytes with greatly improved sensitivity. We applied AmproCode to identify three model peptides including ELA, URP and Aβ. The fmol/L concentration level of the peptide could be detected, illustrating the potential of AmproCode in tracing protein identifications.

Although our initial trials on the synthetic model peptides were successful, we also acknowledge that the labeling inaccuracies, on the proteome level, could not be avoided due to the inherent challenge of the varying reactivities of residues in complex protein chemical environments. This problem might be overcome to a certain extent by the combination of computational simulation and experimental optimization. Given that the statistical labeling rates and off-target rates of a specific reaction on the proteome level could be evaluated from a large number of experimental and simulated samples, an additional calibration program according to these statistical data could be added to our database-matching algorithm to obtain a more accurate and realistic protein identification result [8,24]. Furthermore, during the AmproCode workflow, the proteins can be denatured for DNA barcoding, which could further reduce the complexity of the chemical environments. We envision that our initial success will draw more attention from organic chemists and protein chemists to optimize old reactions and develop new ones that may target more residues with higher selectivity and efficiency. Taken together, additional experimental efforts as well as computational auxiliary tools could improve AmproCode significantly in terms of coverage, accuracy and adaptability.

The current form of AmproCode is only a simplified model for fingerprinting purified or isolated peptides found in the database. In order to analyse complex biological or clinical samples, several other approaches have to be employed to improve the AmproCode method towards a single-molecule technique. Considering the fmol/L level of sensitivity that we have reached, applying more advanced DNA amplification techniques may pave the way for single-molecule AmproCode (Fig. S8). Digital PCR (dPCR) can greatly improve DNA quantitation accuracy at an extremely low concentration [52]. It was reported that dPCR had the capacity to absolutely quantify a level of samples of 0.1–1 copies/μL with reduced experimental variation compared with real-time qPCR [53]. Multiplex PCR is another improvement in residue quantitation because traditional qPCR detects one analyte in one reaction and multiplex PCR allows the quantification of multiple analytes at the same time [54]. We may realize single-protein amplifiable fingerprinting identification in the future with the help of multiplex dPCR. Furthermore, state-of-the-art microfluidic and automated liquid handling techniques are able to isolate diluted samples into microwell chips at the single-molecule level for high-throughput protein identification [55], so additional purification methods could be replaced in AmproCode (Fig. S8). The experimental errors introduced by manual operation during the sample processing workflow could also be reduced.

Taken together, by proteome database-matching for protein identification, the novel concept of amplifiable protein fingerprinting using AmproCode may open a new avenue towards the development of next-generation protein identification and/or sequencing techniques. Our computational and experimental results represent a prototype that could be further developed as a high-throughput method towards single-cell proteomics and the discovery of clinical biomarkers.

## METHODS

### Materials

Peptides were synthesized by GenScript and Hangzhou ALLPEPTIDE Biotechnology. DNA oligos were synthesized by Generay and GenScript. The plasmid was synthesized by GENEWIZ. Fluorescent labeling reagents were purchased from Confluore. DNA modification reagents were purchased from Confluore. Residue-specific peptide modification reagents except Met were purchased from Confluore and 9 Ding Chemistry. The Met specific modification oxaziridine reagent Ox6 was a gift from Prof. Shixian Lin in Zhejiang University. The mobile phases for HPLC including acetonitrile (ACN) and water were purchased from Thermo Fisher Scientific and Wahaha, respectively, and the additives including formic acid, acetic acid, trifluoroacetic acid and triethylamine were purchased from Macklin, J&K Scientific, Energy-Chemical and Thermo Fisher Scientific, respectively. qPCR-related reagents were purchased from YEASEN.

### Database search algorithm

For sequence matching with a target database, ‘cosine similarity’ was chosen as the distance measurement. We implemented a simple python script using the scikit-learn package to calculate the cosine distances from the query sequence to all the sequences within the targeted database by using scipy.spatial.distance.cosine and collected the top *N* results [56]. We manually calculated the ‘cosine similarity’ by using ‘one minus the cosine distance value’ in order to display the results more clearly in this manuscript. Sequences of human proteome were obtained from UniProt release 2021_02 [40]. All known secreted peptide and protein sequences were generated according to ‘PROPEP’ records within the database.

### Theoretical evaluation of the coverage rate

To evaluate the coverage of our AmproCode strategy, we iteratively searched the composition code of each sequence against the whole database. If the query sequence was ranked in the top *N* (*N* = 1 or 5) results, then it was considered as ‘being identified’. The proteome coverage was defined as the percentage of successfully identified proteins. The influence of measurement error was also considered. A Gaussian probability distribution Norm (*μ, σ*^2^) was assigned to all the composition codes in the database, where *μ* was the true value of the composition code and *σ* was the standard deviation of measurements. Thus, using random numbers drawn from Norm (*μ, σ*^2^), we obtained a series of new composition codes that simulated the composition codes with the measurement errors. Then the coverage was calculated as described in the previous paragraph.

### qPCR measurement and data analysis

The qPCR reaction system contained 2 μL of samples, 10 μL of Hieff qPCR SYBR Green Master Mix Low Rox Plus (YEASEN, Cat# 11202ES03), 0.2 μL of the forward primer (10 μM), 0.2 μL of the reverse primer (10 μM) and 7.6 μL of water in the 96-well PCR plate. qPCR was performed on the Applied Biosystems ViiA 7 system (Thermo Fisher Scientific) and the program was as follows: 94°C, 3 min; 32–40 cycles of 94°C, 10 s; 49°C, 20 s; and 72°C, 30 s. In one qPCR measurement, a sample was measured parallelly in three wells on one plate parallelly and the readout mean Ct data were used to calculate one relative ratio. The average of three relative ratios from three independent qPCR measurements was the final result.

## Supplementary Material

nwae183_Supplemental_File

## Data Availability

The relevant code is available on GitHub at https://github.com/wendao/AmproCode-scripts.

## References

[1] Aebersold R, Mann M. Mass-spectrometric exploration of proteome structure and function. Nature 2016; 537: 347–55. 10.1038/nature19949 27629641

[2] Zubarev RA . The challenge of the proteome dynamic range and its implications for in-depth proteomics. Proteomics 2013; 13: 723–6. 10.1002/pmic.201200451 23307342

[3] Shendure J, Balasubramanian S, Church GM et al. DNA sequencing at 40: past, present and future. Nature 2017; 550: 345–53. 10.1038/nature24286 29019985

[4] Grün D, van Oudenaarden A. Design and analysis of single-cell sequencing experiments. Cell 2015; 163: 799–810. 10.1016/j.cell.2015.10.039 26544934

[5] Restrepo-Pérez L, Joo C, Dekker C. Paving the way to single-molecule protein sequencing. Nat Nanotechnol 2018; 13: 786–96. 10.1038/s41565-018-0236-6 30190617

[6] Alfaro JA, Bohländer P, Dai M et al. The emerging landscape of single-molecule protein sequencing technologies. Nat Methods 2021; 18: 604–17. 10.1038/s41592-021-01143-1 34099939 PMC8223677

[7] Swaminathan J, Boulgakov AA, Marcotte EM. A theoretical justification for single molecule peptide sequencing. PLoS Comput Biol 2015; 11: e1004080. 10.1371/journal.pcbi.1004080 25714988 PMC4341059

[8] Swaminathan J, Boulgakov AA, Hernandez ET et al. Highly parallel single-molecule identification of proteins in zeptomole-scale mixtures. Nat Biotechnol 2018; 36: 1076–82. 10.1038/nbt.4278 PMC6482110 30346938

[9] Reed BD, Meyer MJ, Abramzon V et al. Real-time dynamic single-molecule protein sequencing on an integrated semiconductor device. Science 2022; 378: 186–92. 10.1126/science.abo7651 36227977

[10] Brinkerhoff H, Kang ASW, Liu J et al. Multiple rereads of single proteins at single-amino acid resolution using nanopores. Science 2021; 374: 1509–13. 10.1126/science.abl4381 34735217 PMC8811723

[11] Yan S, Zhang J, Wang Y et al. Single molecule ratcheting motion of peptides in a mycobacterium smegmatis porin A (MspA) nanopore. Nano Lett 2021; 21: 6703–10. 10.1021/acs.nanolett.1c02371 34319744

[12] Chen Z, Wang Z, Xu Y et al. Controlled movement of ssDNA conjugated peptide through Mycobacterium smegmatis porin A (MspA) nanopore by a helicase motor for peptide sequencing application. Chem Sci 2021; 12: 15750–56. 10.1039/D1SC04342K 35003607 PMC8654031

[13] Yu L, Kang X, Li F et al. Unidirectional single-file transport of full-length proteins through a nanopore. Nat Biotechnol 2023; 41: 1130–9. 10.1038/s41587-022-01598-3 36624148 PMC10329728

[14] Zhang S, Huang G, Versloot RCA et al. Bottom-up fabrication of a proteasome–nanopore that unravels and processes single proteins. Nat Chem 2021; 13: 1192–9. 10.1038/s41557-021-00824-w 34795436 PMC7612055

[15] Zhang M, Tang C, Wang Z et al. Real-time detection of 20 amino acids and discrimination of pathologically relevant peptides with functionalized nanopore. Nat Methods 2024; 21: 609–18. 10.1038/s41592-024-02208-7 38443507 PMC11009107

[16] Zhang Y, Yi Y, Li Z et al. Peptide sequencing based on host–guest interaction-assisted nanopore sensing. Nat Methods 2024; 21: 102–9. 10.1038/s41592-023-02095-4 37957431

[17] Martin-Baniandres P, Lan W-H, Board S et al. Enzyme-less nanopore detection of post-translational modifications within long polypeptides. Nat Nanotechnol 2023; 18: 1335–40. 10.1038/s41565-023-01462-8 37500774 PMC10656283

[18] Zhao Y, Ashcroft B, Zhang P et al. Single-molecule spectroscopy of amino acids and peptides by recognition tunnelling. Nat Nanotechnol 2014; 9: 466–73. 10.1038/nnano.2014.54 24705512 PMC4047173

[19] Ohshiro T, Tsutsui M, Yokota K et al. Detection of post-translational modifications in single peptides using electron tunnelling currents. Nat Nanotechnol 2014; 9: 835–40. 10.1038/nnano.2014.193 25218325

[20] Hanay MS, Kelber S, Naik AK et al. Single-protein nanomechanical mass spectrometry in real time. Nat Nanotechnol 2012; 7: 602–8. 10.1038/nnano.2012.119 22922541 PMC3435450

[21] Kafader JO, Melani RD, Durbin KR et al. Multiplexed mass spectrometry of individual ions improves measurement of proteoforms and their complexes. Nat Methods 2020; 17: 391–4. 10.1038/s41592-020-0764-5 32123391 PMC7131870

[22] Sungwook W, Yin P. Methods and compositions for protein identification. United States Patent, US2019361031A1.

[23] Hong JM, Gibbons M, Bashir A et al. ProtSeq: toward high-throughput, single-molecule protein sequencing via amino acid conversion into DNA barcodes. iScience 2022; 25: 103586. 10.1016/j.isci.2021.103586 35005536 PMC8717419

[24] de Lannoy CV, Filius M, van Wee R et al. Evaluation of FRET X for single-molecule protein fingerprinting. iScience 2021; 24: 103239. 10.1016/j.isci.2021.103239 34729466 PMC8546410

[25] Shrestha P, Yang D, Tomov TE et al. Single-molecule mechanical fingerprinting with DNA nanoswitch calipers. Nat Nanotechnol 2021; 16: 1362–70. 10.1038/s41565-021-00979-0 34675411 PMC8678201

[26] van Ginkel J, Filius M, Szczepaniak M et al. Single-molecule peptide fingerprinting. Proc Natl Acad Sci USA 2018; 115: 3338–43. 10.1073/pnas.1707207115 29531063 PMC5879649

[27] Püntener S, Rivera-Fuentes P. Single-molecule peptide identification using fluorescence blinking fingerprints. J Am Chem Soc 2023; 145: 1441–7. 10.1021/jacs.2c12561 36603184 PMC9853850

[28] Sano T, Smith CL, Cantor CR. Immuno-PCR: very sensitive antigen detection by means of specific antibody-DNA conjugates. Science 1992; 258: 120–2. 10.1126/science.1439758 1439758

[29] Wik L, Nordberg N, Broberg J et al. Proximity extension assay in combination with next-generation sequencing for high-throughput proteome-wide analysis. Mol Cell Proteomics 2021; 20: 100168. 10.1016/j.mcpro.2021.100168 34715355 PMC8633680

[30] deGruyter JN, Malins LR, Baran PS. Residue-specific peptide modification: a chemist's guide. Biochemistry 2017; 56: 3863–73. 10.1021/acs.biochem.7b00536 28653834 PMC5792174

[31] Reddy NC, Kumar M, Molla R et al. Chemical methods for modification of proteins. Org Biomol Chem 2020; 18: 4669–91. 10.1039/D0OB00857E 32538424

[32] Spicer CD, Davis BG. Selective chemical protein modification. Nat Commun 2014; 5: 4740–53. 10.1038/ncomms5740 25190082

[33] Boutureira O, Bernardes GJL. Advances in chemical protein modification. Chem Rev 2015; 115: 2174–95. 10.1021/cr500399p 25700113

[34] Hoyt EA, Cal PMSD, Oliveira BL et al. Contemporary approaches to site-selective protein modification. Nat Rev Chem 2019; 3: 147–71. 10.1038/s41570-019-0079-1

[35] Lin S, Yang X, Jia S et al. Redox-based reagents for chemoselective methionine bioconjugation. Science 2017; 355: 597–602. 10.1126/science.aal3316 28183972 PMC5827972

[36] Vantourout JC, Adusumalli SR, Knouse KW et al. Serine-selective bioconjugation. J Am Chem Soc 2020; 142: 17236–42. 10.1021/jacs.0c05595 32965106 PMC8350984

[37] Gavrilyuk J, Ban H, Nagano M et al. Formylbenzene diazonium hexafluorophosphate reagent for tyrosine-selective modification of proteins and the introduction of a bioorthogonal aldehyde. Bioconj Chem 2012; 23: 2321–8. 10.1021/bc300410p PMC3526679 23181702

[38] Xie X, Moon PJ, Crossley SWM et al. Oxidative cyclization reagents reveal tryptophan cation–π interactions. Nature 2024; 627: 680–7. 10.1038/s41586-024-07140-6 38448587 PMC11198740

[39] Aspuru-Guzik A, Baik M-H, Balasubramanian S et al. Charting a course for chemistry. Nat Chem 2019; 11: 286–94. 10.1038/s41557-019-0236-7 30903035

[40] Consortium TU . UniProt: the universal protein knowledgebase in 2021. Nucleic Acids Res 2020; 49: D480–9. 10.1093/nar/gkaa1100 PMC7778908 33237286

[41] Uhlén M, Karlsson MJ, Hober A et al. The human secretome. Sci Signal 2019; 12: eaaz0274. 10.1126/scisignal.aaz0274 31772123

[42] Hernandez ET, Swaminathan J, Marcotte EM et al. Solution-phase and solid-phase sequential, selective modification of side chains in KDYWEC and KDYWE as models for usage in single-molecule protein sequencing. New J Chem 2017; 41: 462–9. 10.1039/C6NJ02932A PMC5624723 28983186

[43] Zanon PR, Yu F, Musacchio P et al. Profiling the proteome-wide selectivity of diverse electrophiles. ChemRxiv: 14186561. 10.1038/s41557-025-01902-z PMC12580327 41168333

[44] Hassan SS, Gomez-Lopez N. Reducing maternal mortality: can elabela help in this fight? Lancet North Am Ed 2019; 394: 8–9. 10.1016/S0140-6736(19)30543-4 31282362

[45] Ho L, Dijk M, Chye STJ et al. ELABELA deficiency promotes preeclampsia and cardiovascular malformations in mice. Science 2017; 357: 707–13. 10.1126/science.aam6607 28663440

[46] Georgiadou D, Boussata S, Dijk M. ELABELA measurements by commercial ELISA kits require sample extraction. Am J Physiol Endocrinol Metab 2019; 317: E1218–9. 10.1152/ajpendo.00257.2019 31808726

[47] Timp W, Timp G. Beyond mass spectrometry, the next step in proteomics. Sci Adv 2020; 6: eaax8978. 10.1126/sciadv.aax8978 31950079 PMC6954058

[48] Vaudry H, Leprince J, Chatenet D et al. International union of basic and clinical pharmacology. XCII. Urotensin II, urotensin II–related peptide, and their receptor: from structure to function. Pharmacol Rev 2015; 67: 214–58. 10.1124/pr.114.009480 25535277

[49] Frisoni GB, Altomare D, Thal DR et al. The probabilistic model of Alzheimer disease: the amyloid hypothesis revised. Nat Rev Neurosci 2022; 23: 53–66. 10.1038/s41583-021-00533-w 34815562 PMC8840505

[50] Karran E, De Strooper B. The amyloid hypothesis in Alzheimer disease: new insights from new therapeutics. Nat Rev Drug Discov 2022; 21: 306–18. 10.1038/s41573-022-00391-w 35177833

[51] Podracky CJ, An C, DeSousa A et al. Laboratory evolution of a sortase enzyme that modifies amyloid-β protein. Nat Chem Biol 2021; 17: 317–25. 10.1038/s41589-020-00706-1 33432237 PMC7904614

[52] Heyries KA, Tropini C, VanInsberghe M et al. Megapixel digital PCR. Nat Methods 2011; 8: 649–51. 10.1038/nmeth.1640 21725299

[53] Hindson CM, Chevillet JR, Briggs HA et al. Absolute quantification by droplet digital PCR versus analog real-time PCR. Nat Methods 2013; 10: 1003–5. 10.1038/nmeth.2633 23995387 PMC4118677

[54] Zhong Q, Bhattacharya S, Kotsopoulos S et al. Multiplex digital PCR: breaking the one target per color barrier of quantitative PCR. Lab Chip 2011; 11: 2167–74. 10.1039/c1lc20126c 21584334

[55] Berlanda SF, Breitfeld M, Dietsche CL et al. Recent advances in microfluidic technology for bioanalysis and diagnostics. Anal Chem 2021; 93: 311–31. 10.1021/acs.analchem.0c04366 33170661

[56] Pedregosa F, Varoquaux G, Gramfort A et al. Scikit-learn: machine learning in Python. J Mach Learn Res 2011; 12: 2825–30.

