# Supplementary material for: Amplifiable protein identification via residue-resolved barcoding and composition code counting
Source: Natl Sci Rev. 2024 May 28;11(7):nwae183. doi: 10.1093/nsr/nwae183 (PMC11272068; doi:10.1093/nsr/nwae183)
Supplement: nwae183_Supplemental_File [file nwae183_supplemental_file.docx]

Supplementary data for

**Amplifiable protein identification via residue-resolved barcoding and composition code counting**

Weiming Guo *et al*.

Corresponding authors: Peng R. Chen, pengchen@pku.edu.cn (P. R. C.); Chu Wang, chuwang@pku.edu.cn (C. W.).

**The PDF file includes:**

Methods

Supplementary Figures S1 to S15

Supplementary Tables S1 to S3

References

**Methods**

**Cys specific reaction on small molecules**

(2R)-2-(9H-fluoren-9-ylmethoxycarbonylamino)-3-sulfanylpropanoic acid (Fmoc-L-Cys-OH; bidepharmatech, Cat#BD238587) was dissolved in 15 mM phosphate (PB) buffer pH 6 with 20% (v/v) ACN in a final concentration of 1 mM. N-methyl maleimide (Mal-Me) was added to a final concentration of 5 mM. After 30 min incubation in room temperature, the product was analyzed by the analytical 1260 Infinity II high performance liquid chromatography (HPLC, Agilent) and ACQUITY UPLC I-Class SQD 2 MS spectrometer with electrospray ionization (ESI, Waters).

**Lys specific reaction on small molecules**

(2S)-6-amino-2-(9H-fluoren-9-ylmethoxycarbonylamino)hexanoic acid (Fmoc-L-Lys-OH; Ark Pharm, Cat#AK-41242) was dissolved in 50 mM PB buffer pH 8 with 25% (v/v) ACN in a final concentration of 1 mM. N-acetoxysuccinimide (Ac-NHS, J&K Scientific, Cat#142162) was added to a final concentration of 5 mM. After 30 min incubation in room temperature, the product was analyzed by the analytical 1260 Infinity II HPLC and ACQUITY UPLC I-Class SQD 2 MS spectrometer with ESI.

**Met specific reaction on small molecules**

(2S)-2-(9H-fluoren-9-ylmethoxycarbonylamino)-4-methylsulfanylbutanoic acid (Fmoc-L-Met-OH, Accela, Cat#SY036880) was dissolved in water with 5% (v/v) methanol in a final concentration of 0.1 mM. Ox6 was added to a final concentration of 0.4 mM. After 15 min incubation, the product was analyzed by the analytical Ultimate 3000 UHPLC (Thermo Fisher Scientific) and LCQ Fleet ion trap mass spectrometer (Thermo Fisher Scientific).

**Asp/Glu specific reaction on small molecules**

(4S)-4-(9H-fluoren-9-ylmethoxycarbonylamino)-5-[(2-methylpropan-2-yl)oxy]-5-oxopentanoic acid (Fmoc-L-Glu-OtBu, Ark Pharm, Cat#AK-48532) was dissolved in dimethyl sulfoxide (DMSO) in a final concentration of 1 mM. 2% (v/v) 4-methylmorpholine (NMM, aladdin, Cat#M104643), 50 mM (7-azabenzotriazol-1-yloxy) tripyrrolidinophosphonium hexafluorophosphate (PyAOP, Energy-Chemical, Cat#E020438) and 30 mM methylamine (Hushi, Cat# 80081118) were added to the system. After 2 h incubation in room temperature, the product was analyzed by the analytical 1260 Infinity II HPLC and Ultimate 3000 UHPLC-LCQ Fleet ion trap mass spectrometer.

**Tyr specific reaction on small molecules**

(2S)-2-(9H-fluoren-9-ylmethoxycarbonylamino)-3-(4-hydroxyphenyl)propanoic acid (Fmoc-L-Tyr-OH, Macklin, Cat#F809877) was dissolved in 10mM PB buffer pH 8 with 50% ACN in the concentration of 5 μM. 4-formylbenzene diazonium hexafluorophosphate was added to a final concentration of 25 μM. After 1 h incubation in room temperature, the product was analyzed by the analytical Ultimate 3000 UHPLC and LCQ Fleet ion trap mass spectrometer.

**Cys and Lys specific reactions on peptides**

The model peptide (TAMRA-GAGSKGSAGSDGSCGSAGSMG-CONH_2_) was dissolved in 100 mM PB buffer pH 8 in a final concentration of about 0.5 mM. Mal-Me and Ac-NHS were added to a final concentration of 2 mM in one-pot. After incubation for 30 min in the room temperature, the product was analyzed by the analytical 1260 Infinity II HPLC and ACQUITY UPLC I-Class SQD 2 MS spectrometer with ESI.

The model peptide with Cys and Lys modifications (protections) was purified by semi-preparative 1260 Infinity II HPLC (Agilent) and then freeze-dried for further Met and Asp/Glu specific reactions.

**Met specific reaction on peptides**

The model peptide (TAMRA-GAGSKGSAGSDGSCGSAGSMG-CONH_2_) with Cys and Lys protections was dissolved in water with 5% (v/v) MeOH in a final concentration of 0.5 mM. Ox6 was added to a final concentration of 2 mM. After incubation for 15 min in the room temperature, the product was analyzed by the analytical 1260 Infinity II HPLC and ACQUITY UPLC I-Class SQD 2 MS spectrometer with ESI.

**Asp/Glu specific reaction on peptides**

The model peptide (TAMRA-GAGSKGSAGSDGSCGSAGSMG-CONH_2_) with Cys and Lys protection was dissolved in DMSO with 10% (v/v) water in a final concentration of about 0.5 mM. 2% NMM, 70 mM PyAOP and 30 mM n-butylamine were added to the system. After incubation for 2 h in the room temperature, the product was analyzed by the analytical 1260 Infinity II HPLC and ACQUITY UPLC I-Class SQD 2 MS spectrometer with ESI.

**DNA modification and purification**

The DNA with the 5’ end amine modification was dissolved in 100 mM PB buffer pH 8 with 10% (v/v) DMSO in a final concentration of 0.5 to 1 mM. The NHS reagents including Mal-PEG6-NHS (Confluore, Cat#BPM-35), Mal-PEG12-NHS (9DingChem, Cat#L-FV438), DBCO-PEG4-NHS (Confluore, Cat#BCD-88) and DBCO-PEG12-NHS (Confluore, Cat#BCD-9) were respectively added to a final concentration of 5 mM in order to attach the Mal or dibenzocyclooctyne (DBCO) group to DNA (Mal-DNA or DBCO-DNA for short). After 40 min reaction in room temperature, the modified DNA was purified by ethanol precipitation. The DNA was dissolved in water and was quantified by NanoDrop microvolume spectrophotometers and fluorometer (Thermo Fisher Scientific) and Ultimate 3000 UHPLC- LCQ Fleet ion trap mass spectrometer.

**Cys specific DNA barcoding on ELA and URP**

The peptide was dissolved in 20 mM phosphate (PB) buffer pH 6 in a final concentration of 1 mM. The Mal-DNA was added 3 times more than the concentration of Cys residues. After 30 min incubation in the room temperature, the DNA peptide conjugate was purified by HPLC and identified by Ultimate 3000 UHPLC- LCQ Fleet ion trap mass spectrometer or Quadrupole-TOF LC-MS System (Waters) in Analysis and Testing Center of Peking University.

**Lys-specific DNA barcoding on ELA and URP**

The peptide was dissolved in 20 mM PB buffer pH 6 in the concentration of 1 mM. The peptide was incubated with 10 mM N-methylmaleimide for 30 min in the room temperature and the purified by HPLC. The freeze-dried sample was dissolved in 100 mM PB buffer pH 8 in the concentration of less than 1 mM and was incubated with 10 mM thiolester or azide modified NHS ester (AcS-PEG4-NHS ester, Confluore, Cat#BHT-9 or azido-PEG4-NHS ester, Confluore, Cat#BPA-10) for 30 min in the room temperature. The functionalized peptide was purified by semi-preparative 1260 Infinity II HPLC and then freeze dried. For thioester functionalized peptide, it was dissolved in 20 mM PB buffer pH 6 and pretreated by 60 mM hydroxylamine in room temperature for 30 min. The Mal-DNA was added 4 times more than the concentration of Lys residues for 30 min incubation in the room temperature. For azide functionalized peptide, it was dissolved in 20 mM PB buffer pH 7 and the DBCO-DNA was added 4 times more than the concentration of Lys residues for 2 h incubation in the room temperature. The DNA peptide conjugate was purified by semi-preparative 1260 Infinity II HPLC and identified by Ultimate 3000 UHPLC- LCQ Fleet ion trap mass spectrometer or Quadrupole-TOF LC-MS System in Analysis and Testing Center of Peking University.

**Met-specific DNA barcoding on ELA**

The peptide was dissolved in 100 mM PB buffer pH 8 in the concentration of 1 mM. The peptide was incubated with 10 mM Mal-Me and 10 mM Ac-NHS for 30 min in the room temperature and then purified by semi-preparative 1260 Infinity II HPLC. The freeze-dried sample was dissolved in 10 mM PB buffer pH 7 for Met functionalization. Ox6 was added about 3 times more than Met residues. After 15 min incubation in the room temperature, the functionalized peptide was purified by semi-preparative 1260 Infinity II HPLC and then freeze dried. The peptide was dissolved in 10 mM PB buffer pH 7 and the DBCO modified DNA was added 4 times more than the concentration of Met residues. After 2 h incubation in the room temperature, the DNA peptide conjugate was purified by semi-preparative 1260 Infinity II HPLC and identified by Ultimate 3000 UHPLC- LCQ Fleet ion trap mass spectrometer or Quadrupole-TOF LC-MS in Analysis and Testing Center of Peking University System.

URP has no Met residue, so Ox6 cannot react with URP.

**Asp/Glu/C-terminal-specific DNA barcoding on ELA and URP**

The peptide was dissolved in 100 mM PB buffer pH 8 in the concentration of 1 mM. The peptide was incubated with 10 mM Mal-Me and 10 mM Ac-NHS for 30 min in the room temperature to protect Cys and Lys and then purified by semi-preparative 1260 Infinity II HPLC. The freeze-dried sample was dissolved in N, N-dimethylformamide (DMF) or DMSO with 10% (v/v) water in the concentration of less than 0.5 mM. Then 2% (v/v) 4-Methylmorpholine, 50 mM PyAOP and 50 mM azido-PEG3-amine (Confluore, Cat#BAPA-3) were added to the sample and incubated for 2 h in the room temperature. The functionalized peptide was purified by semi-preparative 1260 Infinity II HPLC and then freeze dried. The sample was dissolved in 10 mM PB buffer pH 7 and the DBCO modified DNA was added 4 times more than the concentration of Asp and Glu residues as well as the C-terminal. After 2 h incubation in the room temperature, the DNA peptide conjugate was purified by semi-preparative 1260 Infinity II HPLC and identified by Ultimate 3000 UHPLC- LCQ Fleet ion trap mass spectrometer or Quadrupole-TOF LC-MS in Analysis and Testing Center of Peking University System.

**Tyr-specific DNA barcoding on URP**

The peptide was dissolved in 100 mM PB buffer with 50% (v/v) DMSO pH 8 in the concentration of 0.5 mM. The peptide was incubated with 10 mM Mal-Me and 10 mM Ac-NHS for 30 min in the room temperature to protect Cys and Lys. The supernatant was collected by centrifugation and purified by semi-preparative 1260 Infinity II HPLC. The freeze-dried sample was dissolved in 10 mM PB buffer pH 8 with 30% (v/v) ACN and the formylbenzene diazonium reagent was added 2 times more than the concentration of Tyr for 45 min incubation. Then the azide modified hydroxylamine (Aminooxy-PEG4-azide, Confluore, Cat#BPA-44) was added 2 times more than the concentration of formylbenzene diazonium and pH was adjusted to 6 for another 2 h incubation. The Tyr functionalized peptide was purified by semi-preparative 1260 Infinity II HPLC and then freeze dried. The peptide was dissolved in 10 mM PB buffer pH 7 and the DBCO modified DNA was added 4 times more than the concentration of Tyr. After 2 h incubation in the room temperature, the DNA peptide conjugate was purified by semi-preparative 1260 Infinity II HPLC and identified by Ultimate 3000 UHPLC- LCQ Fleet ion trap mass spectrometer.

**SrtAβ mediated Aβ modification**

The synthetic Aβ_40_ peptide were supplemented in a final concentration of 150 μM with 30 mM of NaCl, 5 mM of CaC_l2_ and 15 mM of PB buffer pH 8 and 50 μM of SrtAβ and 1.2 mM of GGGK(TAMRA). To imitate a complex sample, we also added the other peptide including 0.5 mM Humanin-like 9 (HMN-9), 1 mM URP and 0.5 mM NY-ESO-1 (157-165) antigen peptide. The reaction was quenched by adding 1% formic acid after 2 hours. They were analyzed by ACQUITY UPLC I-Class SQD 2 MS spectrometer with ESI. The product was purified by semi-preparative 1260 Infinity II HPLC.

**Aβ related DNA barcoding**

i) Lys-specific DNA barcoding. The Aβ-TAMRA peptide was dissolved in the concentration of about 0.1 mM in 20 % 10 mM PB buffer pH 8 and 80% DMSO with 0.1% triethylamine. The peptide was reacted with 2mM AcS-PEG4-NHS ester for 40 min in the room temperature. The functionalized peptide was purified by semi-preparative 1260 Infinity II HPLC and then freeze dried. After that, it was dissolved in 20 mM PB buffer pH 6 and pretreated by 100 mM hydroxylamine in room temperature for 30 min. The Mal-DNA was added 5 times more than the concentration of Lys residues for 30 min incubation in the room temperature. The DNA peptide conjugate was purified by semi-preparative 1260 Infinity II HPLC and identified by Ultimate 3000 UHPLC- LCQ Fleet ion trap mass spectrometer.

ii) Met-specific DNA barcoding. The Aβ-TAMRA peptide was dissolved in 10 mM PB buffer pH 8 in the concentration of 0.5mM and Ox6 was added about 3 times more than Met residues. After 15 min incubation in the room temperature, the functionalized peptide was purified by semi-preparative 1260 Infinity II HPLC and then freeze dried. Then it was dissolved in 10 mM PB buffer pH 7 and the DBCO modified DNA was added 4 times more than the concentration of Met residues. After 2 h incubation in the room temperature, the DNA peptide conjugate was purified by semi-preparative 1260 Infinity II HPLC and identified by Ultimate 3000 UHPLC- LCQ Fleet ion trap mass spectrometer.

iii) Tyr-specific DNA barcoding. The Aβ-TAMRA peptide was dissolved in 10 mM PB buffer pH 8 with 20% (v/v) DMSO in the concentration of 0.5 mM. The formylbenzene diazonium reagent was added 2 times more than the concentration of Tyr for 45 min incubation. Then the aminooxy-PEG4-azide was added 2 times more than the concentration of formylbenzene diazonium and pH was adjusted to 6 for another 2 h incubation. The Tyr functionalized peptide was purified by semi-preparative 1260 Infinity II HPLC and freeze dried. The peptide was dissolved in 10 mM PB buffer pH 7 and the DBCO modified DNA was added 4 times more than the concentration of Tyr. After 2 h incubation in the room temperature, the DNA peptide conjugate was purified by semi-preparative 1260 Infinity II HPLC and identified by Ultimate 3000 UHPLC- LCQ Fleet ion trap mass spectrometer.

iv) Aβ peptide has no Cys residue so Mal-DNA cannot be attached to Aβ-TAMRA in the condition of 20 mM PB buffer pH 6.

**Mass spectrometry analysis**

The ACQUITY UPLC I-Class SQD 2 ESI MS spectrometer equipped with the ACQUITY UPLC BEH C18 column 130Å 1.7 µm 2.1×50 mm (Waters, Cat# 186002350) was applied for small molecule analysis and peptide analysis. For the small molecule reaction analysis, the mobile phases were water and acetonitrile, the gradient was from 5% to 100% acetonitrile in 3.4 min and the flow rate was 0.3 mL/min. For the peptide analysis, 0.1% formic acid was added to both mobile phases and the gradient was from 5% to 100% acetonitrile in 4.1 min with the same flow rate. The Ultimate 3000 UHPLC- LCQ Fleet ion trap mass spectrometer equipped with the Kinetex 2.6 μm C18 100 Å LC column 50×3 mm (Phenomenex, Cat#00B-4462-Y0) and Clarity 2.6 μm Oligo-MS 100A LC column 50×2.1 mm (Phenomenex, Cat#00B-4479-AN) was applied for the small molecule analysis and the DNA-peptide conjugate analysis respectively. The mobile phases for small molecule analysis were water and acetonitrile and the gradient was from 5% to 100% acetonitrile in 5 min with the flow rate of 0.8 mL/min. For DNA-peptide conjugate analysis, 10 mM triethylamine acetate (TEAA) buffer pH 5.5 and acetonitrile were chosen as the mobile phases, the gradient was from 5% to 50% acetonitrile in 5.5 min with the flow rate of 0.4 mL/min. Some ELA related DNA-peptide conjugate samples were analyzed by Quadrupole-TOF LC-MS System (Waters) at the Analysis and Testing Center of Peking University. The mobile phases and gradient were similar. All the spectra data were exported and analyzed using Origin 2020b.

**Fluorescence calibration**

In fluorescence calibration, the TAMRA-fluorescent standard peptide was dissolved in the water in the concentration of 1 mM as a standard sample. Then it was diluted to 1 μM by 10 mM Tris pH 8 with 10% (v/v) DMSO and then diluted twice gradually from 0.5 μM to 0.031 μM. The samples were also diluted in 10 mM Tris pH 8 with 10% (v/v) DMSO or 10 mM PB buffer pH 8 with 40% (v/v) ACN (for URP peptide). After pipetting samples into the 384 well black plate, the concentration of the samples was quantified by the fluorescence measurement (excitation 555 nm, emission 585 nm) by the Multi-Mode Microplate Reader (BioTek). Finally, we diluted the samples to 1.67 nM and stored at -80 ℃ for further usage.

**A260 quantitation**

In A260 quantitation, the 260 nm absorbance of samples was measured by NanoDrop microvolume spectrophotometers and fluorometer. Using the molecule weight of DNA, the concentration of the samples could be calculated. We diluted the samples to 1.67 nM (10^9^ copies/μL) and stored at -80 °C for further usage if necessary.

**Expression and purification of SrtAβ**

The expression and purification of SrtAβ has already been reported [1].The pET29 plasmid for SrtAβ expression was transformed into *E. coli* BL21(DE3). Cells were cultured at 37 °C in 2× YT medium with 50 μg/ml of kanamycin until OD600 reached 0.6. IPTG was added to a final concentration of 1 mM and protein expression was induced overnight at 25 °C. After collecting by centrifugation at 3000 g for 30 min, they were resuspended in PBS and lysed by sonication (2s on, 2s off for 30 min). The sample was centrifugated at 20000 g at 4 °C for 30 min and the supernatant was purified on a Ni-NTA column (YEASEN, Cat# 20504ES08) and a desalting column (YEASEN, Cat# 20590ES03). The enzyme concentration was quantified by NanoDrop using A280 and stored in -30 °C after adding 10% (v/v) glycerol.

**Synthesis of 4-formylbenzene diazonium hexafluorophosphate**

The synthetic procedure of 4-formylbenzene diazonium hexafluorophosphate has already been reported in previous work [2]. 4-aminobenzaldehyde (500 mg, 4.13 mmol, Accela, Cat# SY003470) was suspended in 6N HCl (6 mL) and cooled at 0 ℃. The solution of NaNO_2_ (330 mg, 4.78 mmol) in water (4 mL) was slowly added to it and the resulting solution was stirred at -0 ℃ for 30 min. Then the precooled 60% HPF_6_ (1.25 mL in water, 5 mmol, Aladdin, Cat#H105677) was and stirred added at -0 ℃ for another 40 min. The resulting solid was collected by filtration, washed by cool water for 3 times and freeze-dried to give the product as a yellow solid (570 mg, 2.05 mmol, 49.6%).


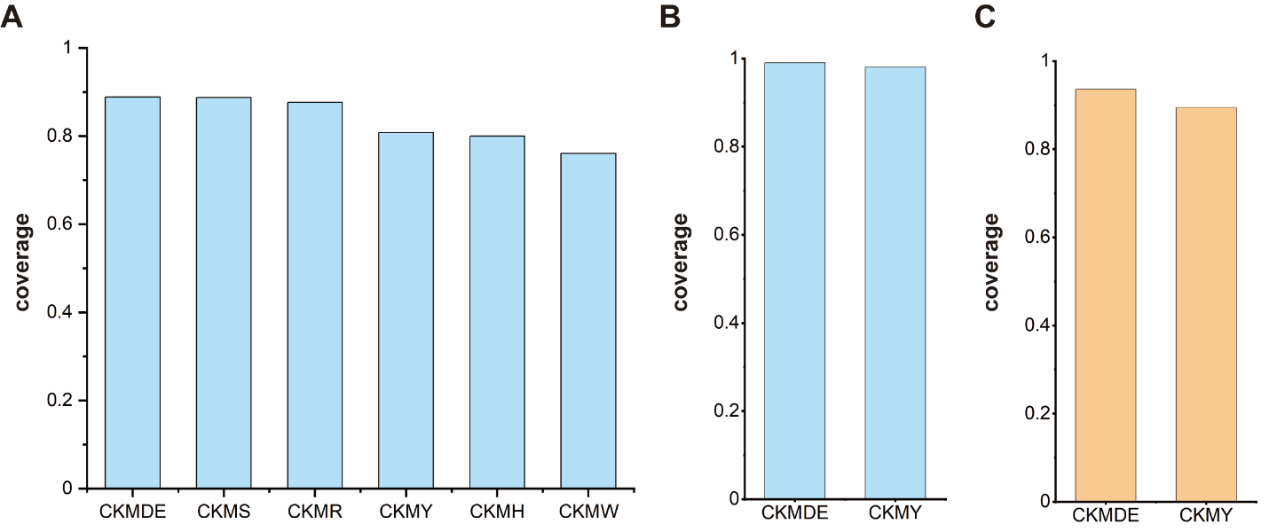


**Supplementary Figure S1. Theoretical evaluation of the coverage rate of AmproCode using different composition codes.** (A) The identification rate of AmproCode (top 1) in the whole human proteome using different combinations of four residues. The combination contains Cys, Lys, Met, Asp/Glu; Cys, Lys, Met, Ser; Cys, Lys, Met, Arg; Cys, Lys, Met, Tyr; Cys, Lys, Met, His; as well as Cys, Lys, Met, Trp. (B) The coverage rate of AmproCode (top 5) in the whole human proteome using the composition code of Cys, Lys, Met, Asp/Glu or Cys, Lys, Met, Tyr. (C) The coverage rate of AmproCode (top 5) in the secretome using the composition code of Cys, Lys, Met, Asp/Glu or Cys, Lys, Met, Tyr.


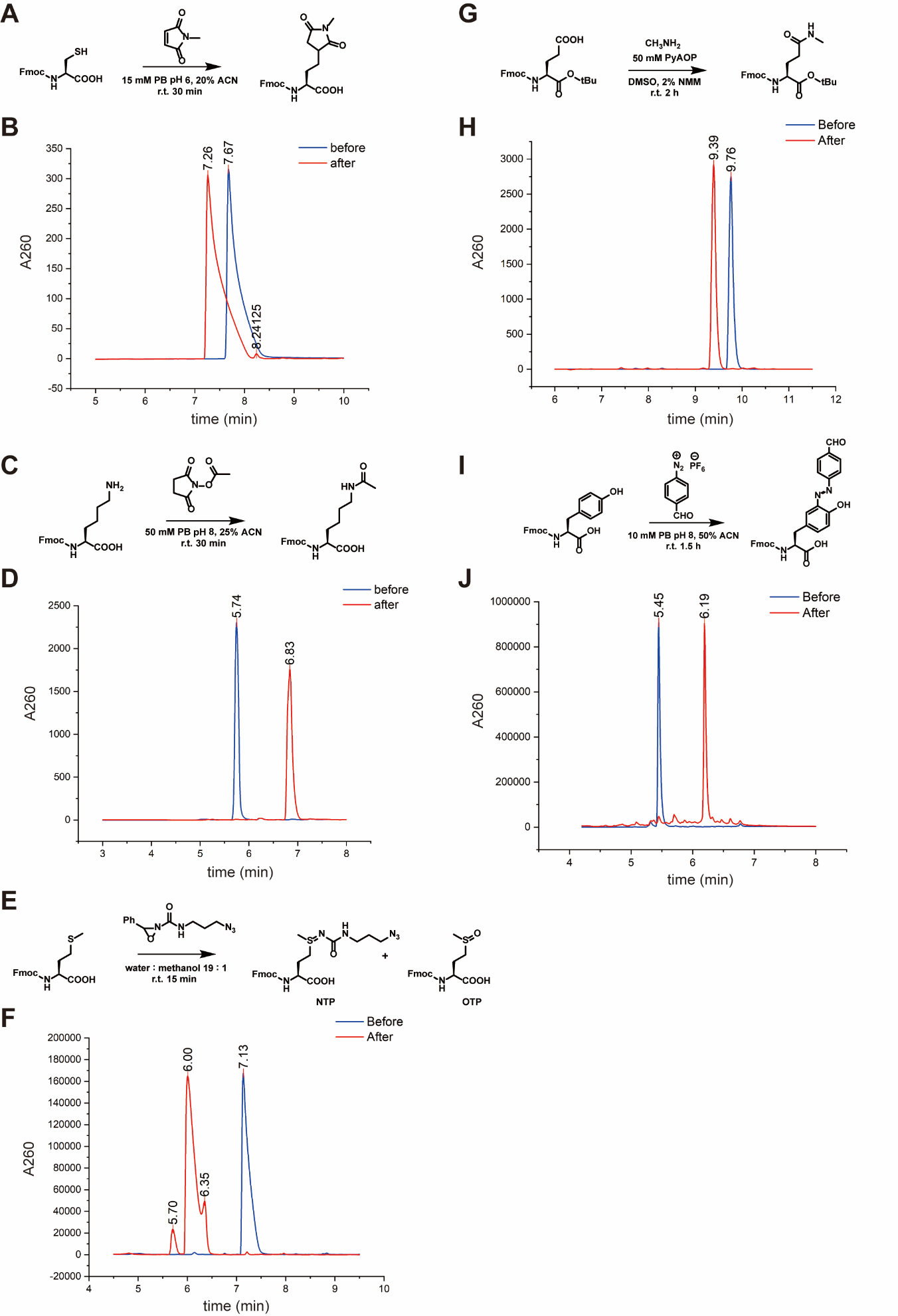


**Supplementary Figure S2. Quantitation of residue specific modification on the amino acids.** (A-B) Cys-specific modification. (A) The reaction scheme of Cys-specific modification. (B) A260 traces of the sample before (blue) and after (red) the reaction in HPLC. The reaction yield is quantified as 99.3% by the peak area. (C-D) Lys-specific modification. (C) The reaction scheme of Lys-specific modification. (D) A260 traces of the sample before (blue) and after (red) the reaction in HPLC. The reaction is quantitative. (E-F) Met-specific modification. (E) The reaction scheme of Met-specific modification. (F) A260 traces of the sample before (blue) and after (red) the reaction in HPLC. The conversion rate of the Fmoc-Met-OH is over 99.6% with the ratio of NTP : OTP = 13.5 : 1 (the peak at 6.00 is the NTP product and the peak at 5.70 min is the OTP by-product). The peak at 6.35 min is decomposed Ox6 after reaction. (G-H) Asp/Glu-specific modification. (G) The reaction scheme of Glu-specific modification. (H) A260 traces of the sample before (blue) and after (red) the reaction in HPLC. The reaction is quantitative. (I-J) Tyr-specific modification. (I) The reaction scheme of Tyr-specific modification. (J) A260 traces of the sample before (blue) and after (red) the reaction in HPLC. The peak at 6.19 min is the product. The peak at 5.45 is the reactant Fmoc-Tyr-OH. The conversion rate of the Fmoc-Tyr-OH is quantified as 92.6% by the peak area.


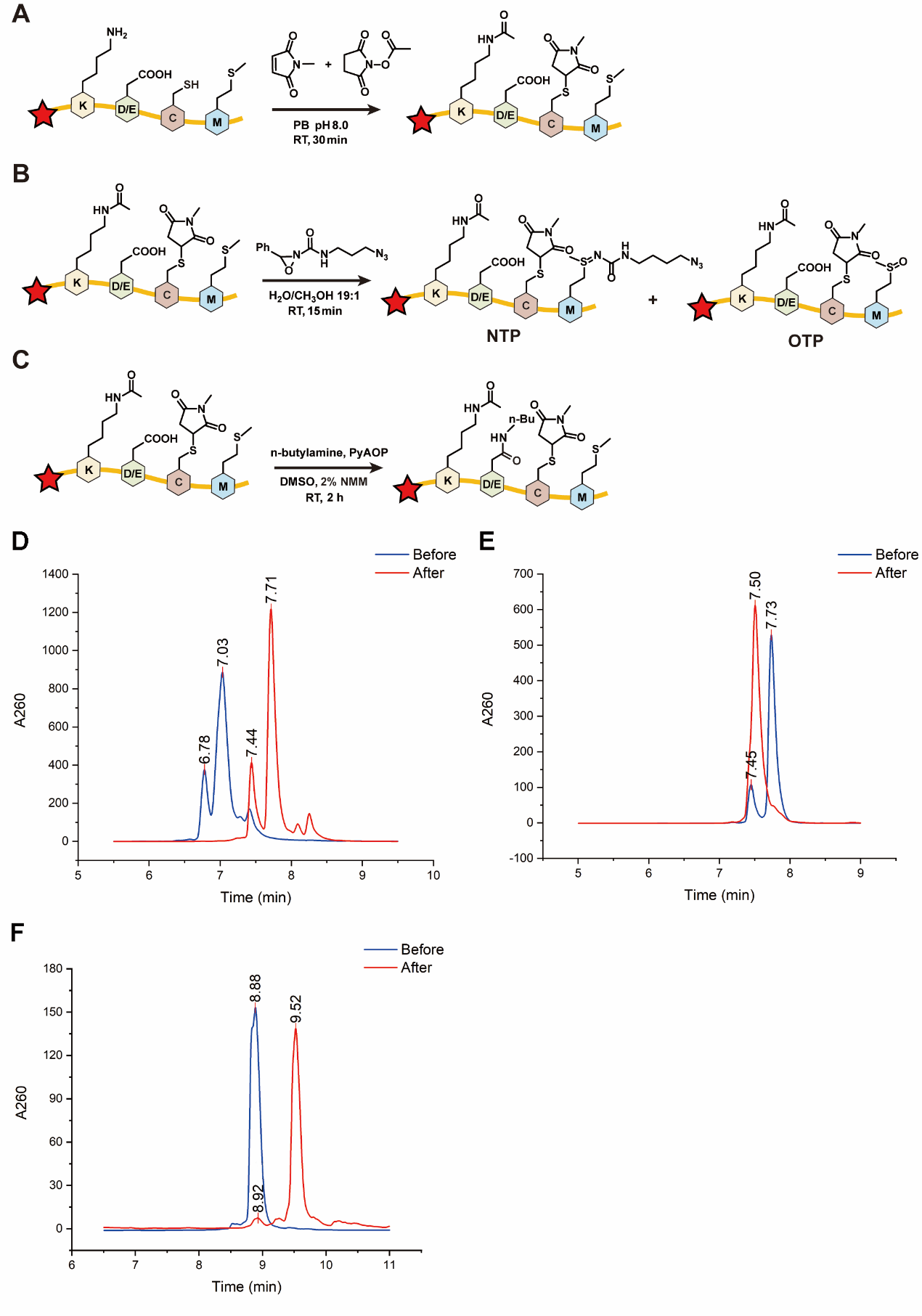


**Supplementary Figure S3. Quantitation of residue-specific reactions on the model peptide.** (A) The reaction scheme of the one-pot Cys-specific reaction and Lys-specific reaction. (B) The reaction scheme of Met-specific modification. (C) The reaction scheme of Asp/Glu-specific modification. (D) A260 traces of the sample before (blue) and after (red) the one-pot Cys-specific reaction and Lys-specific reaction in HPLC. The reaction is quantitative. (E) A260 traces of the sample before (blue) and after (red) the Met-specific modification reaction in HPLC. The main product (NTP) is mixed with the little by-product (OTP) in one peak at 7.50. The conversion of the reactant is nearly complete. (F) A260 traces of the sample before (blue) and after (red) the Asp-specific modification reaction in HPLC. The conversion rate is quantified as 95.1% by the peak area.


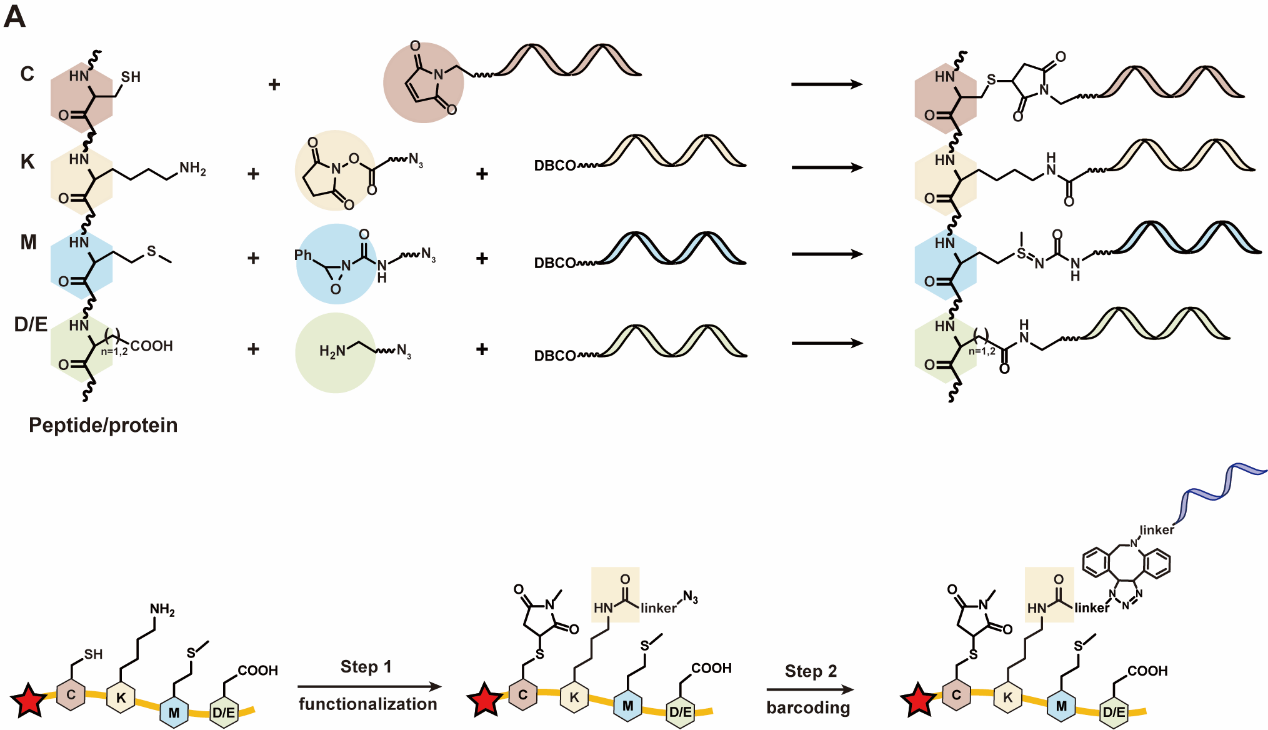


**Supplementary Figure S4. A two-step Lys-specific DNA barcoding procedure based on the SPAAC reaction between azide and DBCO.** In the first functionalization step, some sensitive amino acids are protected and the azide group is attached to Lys. In the second barcoding step, it is labeled by DBCO-DNA.


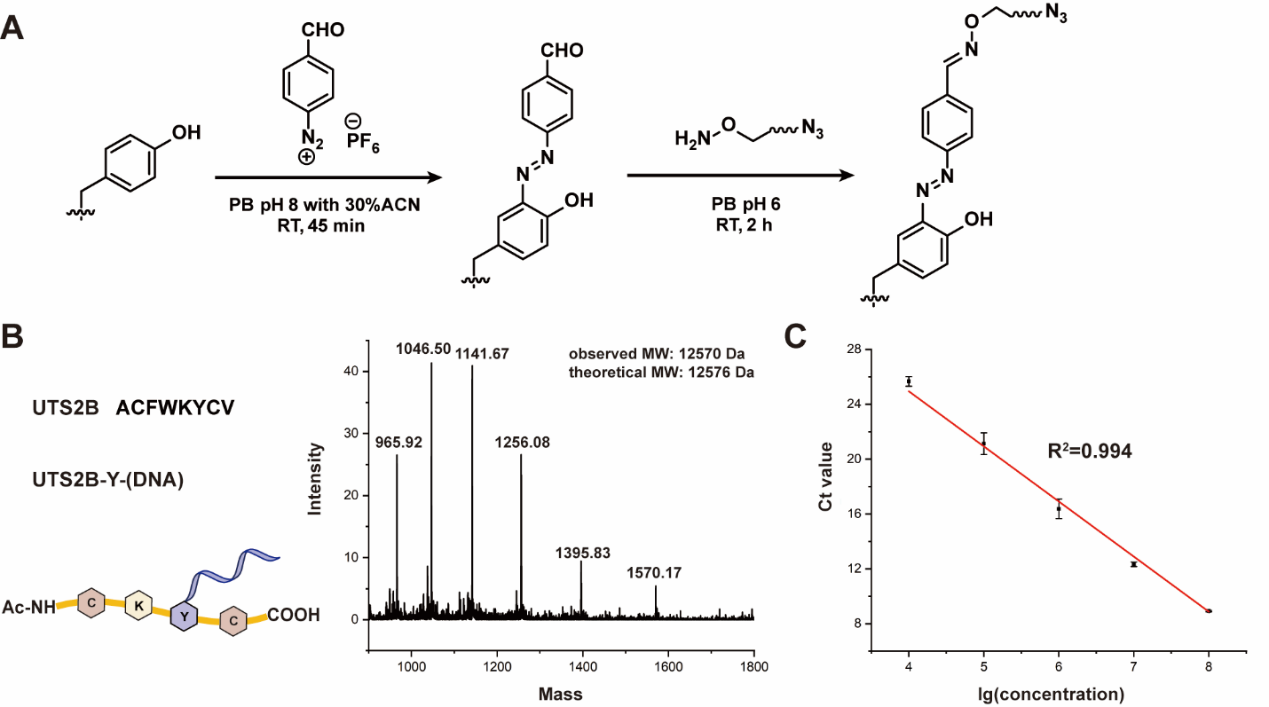


**Supplementary Figure S5. Tyr-specific DNA barcoding on peptides.** (A) Scheme of the Tyr-specific reaction in one-pot. (B) Structure and full mass spectrum of Tyr-specific DNA barcoded URP, URP-Y-(DNA). The theoretical molecule weight (MW) is 12576 Da and the observed MW is 12570 Da. (C) qPCR performance of URP-Y-(DNA). The linear range is from 10^8^ to 10^4^ copies/μL (R^2^=0.994).


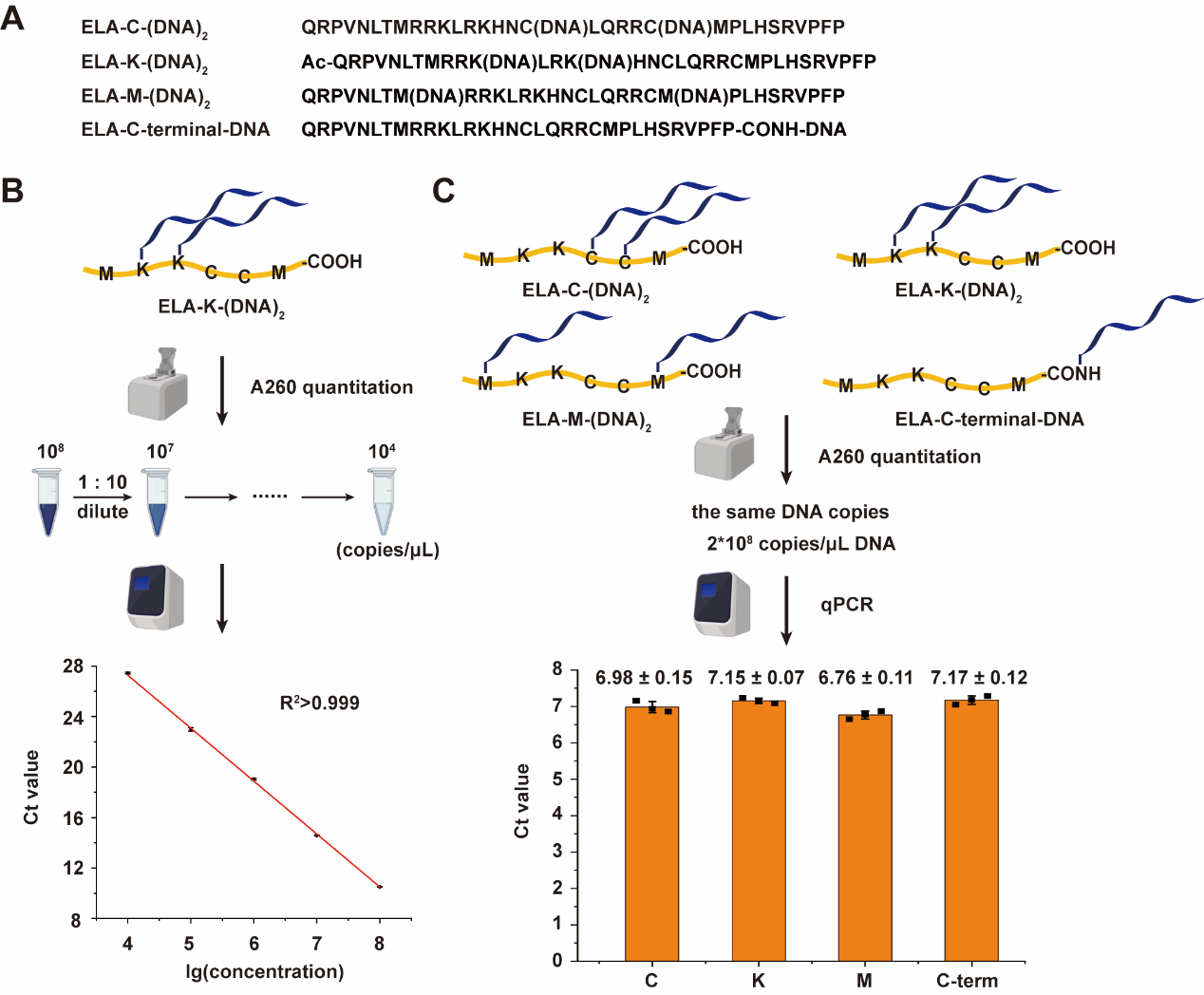


**Supplementary Figure S6. qPCR performance of the DNA barcoded peptides.** (A) The sequence of the DNA barcoded peptides including ELA-C-(DNA)_2_, ELA-K-(DNA)_2_, ELA-M-(DNA)_2_ and ELA-C-terminal-DNA. (B) After quantitation of the DNA concentration of by the absorbance in 260, ELA-K-(DNA)_2_ is diluted from 10^8^ to 10^4^ copies/μL to performance qPCR. The linear correlation of the qPCR from 10^8^ to 10^4^ copies/μL is excellent (R^2^>0.999). (C) qPCR readout between the four samples after normalization of DNA copies by the absorbance in 260 nm. In the concentration of 2*10^8^ copies/μL of DNA, similar Ct values are readout by qPCR which are 6.98±0.15 (ELA-C-(DNA)_2_), 7.15±0.07 (ELA-K-(DNA)_2_), 6.76±0.11 (ELA-M-(DNA)_2_) and 7.17±0.12 (ELA-C-terminal-DNA).


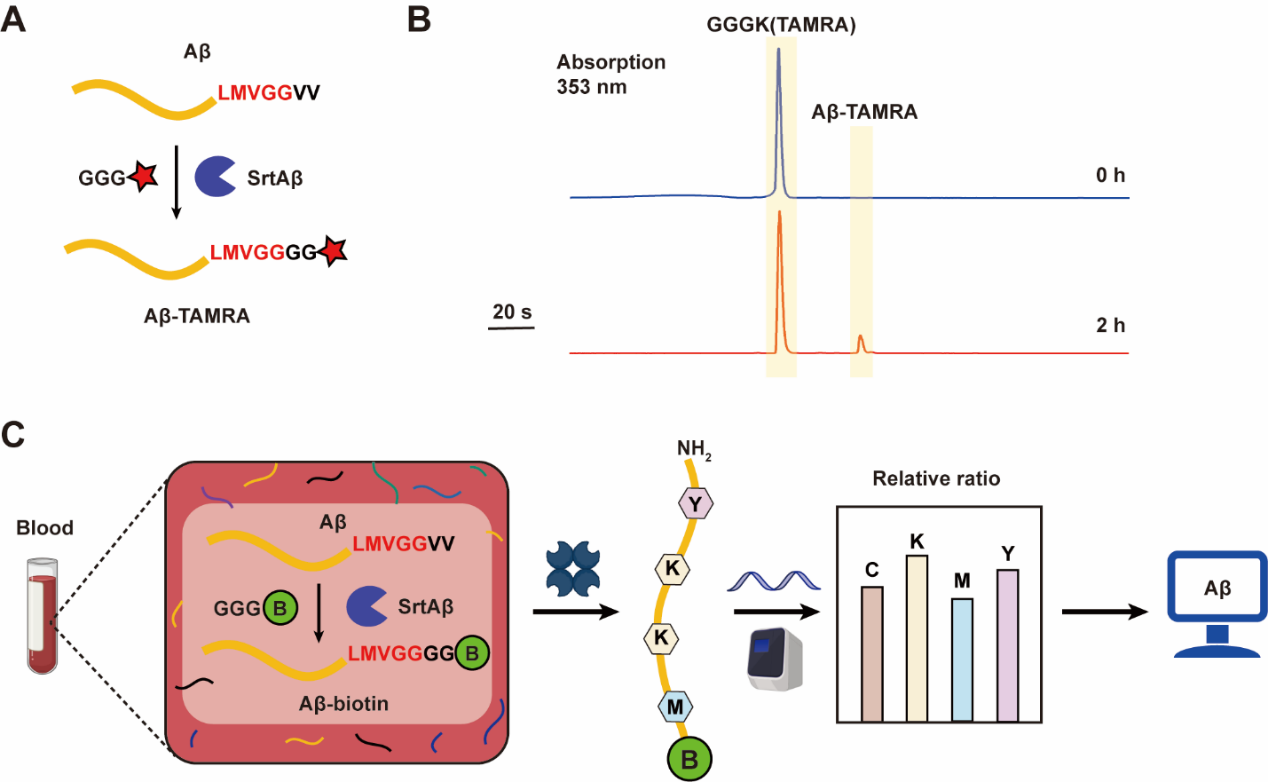


**Supplementary Figure S7. SrtAβ mediated Aβ modification.** (A) Reaction of Aβ-specific modification by SrtAβ. SrtAβ recognize the LMVGG sequence near the C-terminal of the Aβ peptide and it can attach a functional group after it such as the TAMRA fluorescent dye. (B) A353 traces of the reaction sample in HPLC at 0 h (blue, up) and 2 h (red, bottom). (C) A possible strategy to enrich Aβ peptide from clinical blood samples for AmproCode identification. SrtAβ-mediated biotin modification helps to enrich Aβ peptide by streptavidin from clinical blood samples. After DNA modification and qPCR amplification, trace Aβ peptide can be identified via AmproCode.


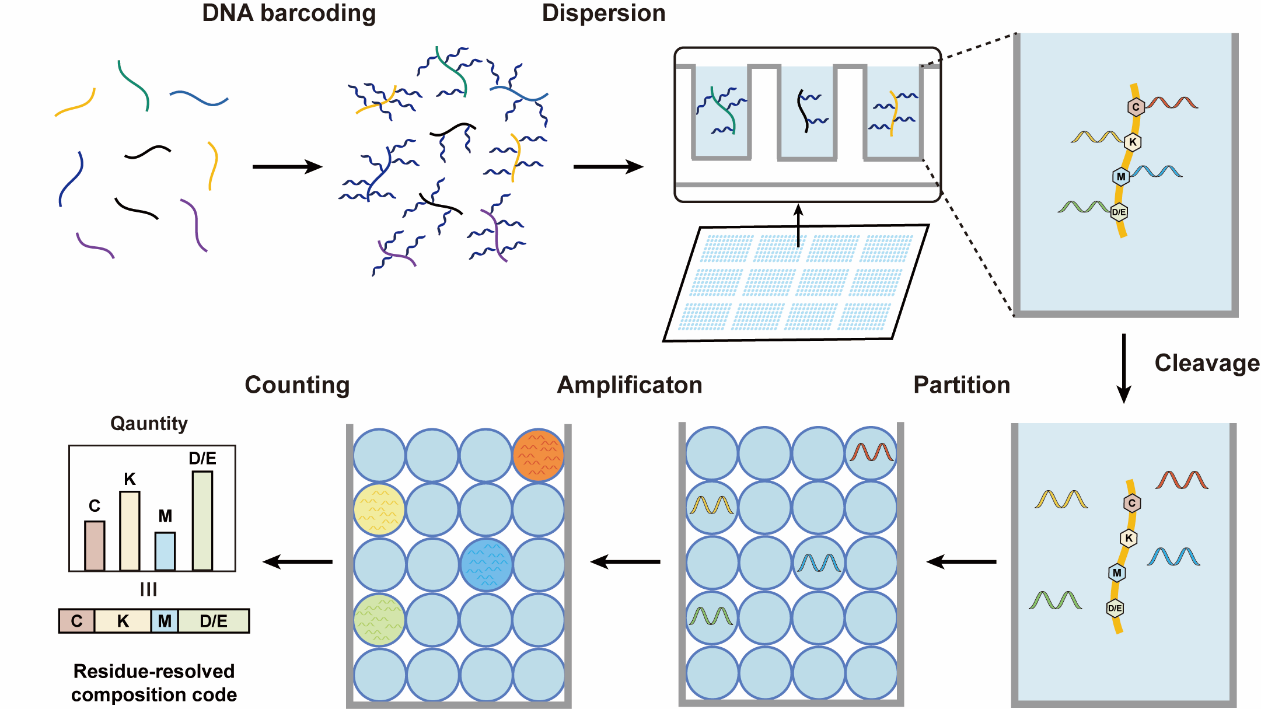


**Supplementary Figure S8. An envisioned scheme of single-molecule AmproCode based on multiplex dPCR and the high-throughput microwell plate.** The protein samples are labeled with residue-specific DNA barcodes containing cleavable linkers. Then, the labeled proteins are dispersed in the microwell plate at single-molecule level. The DNA barcodes cleaved from the protein through the cleavage reactions, and the solution in each well is partitioned into multiple droplets. After amplification via multiplex PCR, the droplets containing DNA barcodes can be detected by the corresponding fluorescence. Poisson statistical analysis of the droplets gives the residue-resolved composition code of the protein.


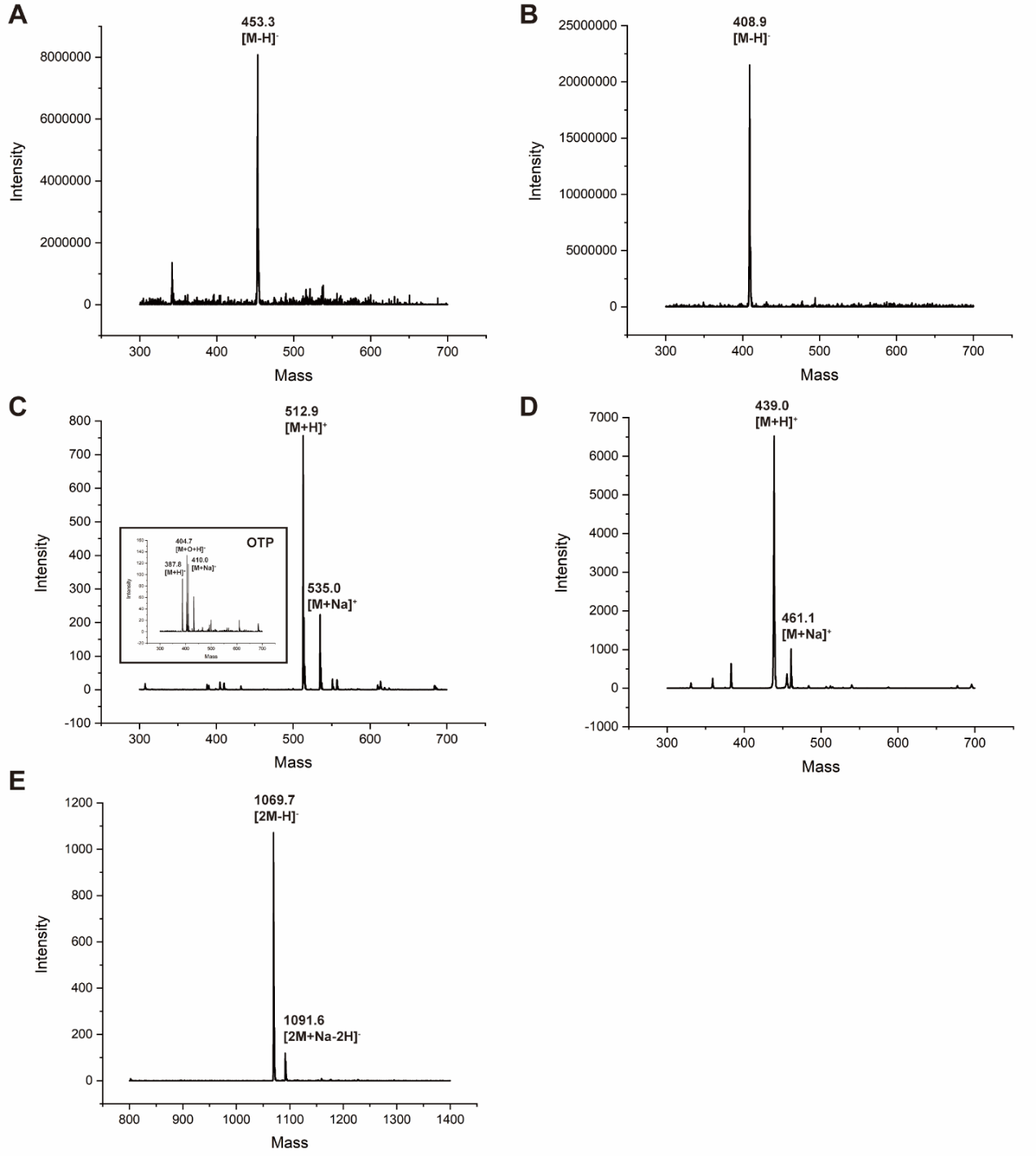


**Supplementary Figure S9. Mass spectra of the small molecules after residue-specific modification.** (A) Full mass spectrum of the expected modified Fmoc-Cys-OH ([M-H]^-^ ion) with m/z of 453.3. (B) Full mass spectrum of the expected modified Fmoc-Lys-OH ([M-H]^-^ ion) with m/z of 408.9. (C) Full mass spectra of the modified Fmoc-Met-OH. The expected NTP main product ([M+H]^+^ ion) is with m/z of 512.9 and the OTP by-product ([M+H]^+^ ion) is with m/z of 387.8 (in square). (D) Full mass spectrum of the expected modified Fmoc-Glu-OtBu ([M+H]^+^ ion) with m/z of 439.0. (E) Full mass spectrum of the expected modified Fmoc-Tyr-OH ([2M-H]^-^ ion) with m/z of 1069.7.


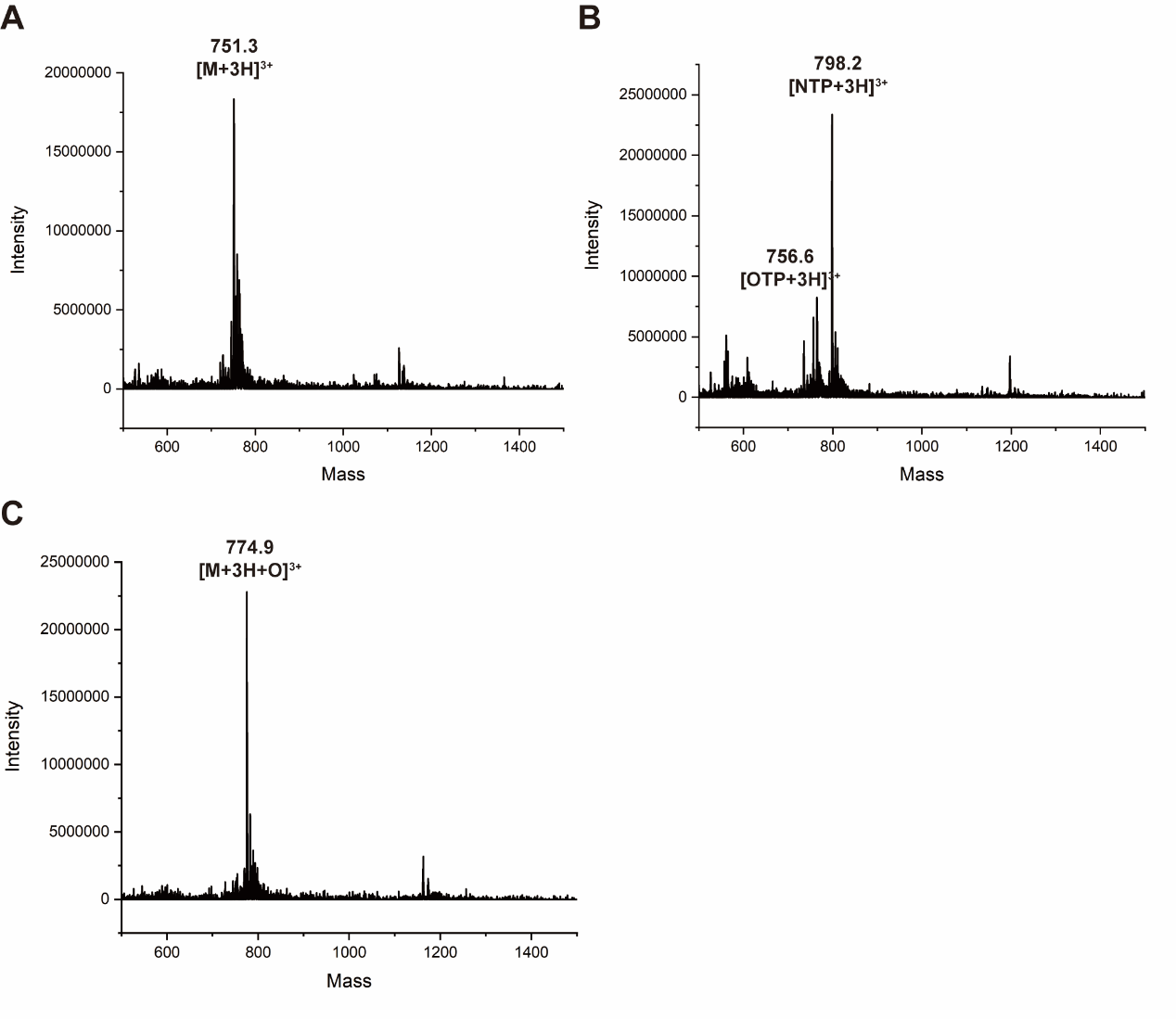


**Supplementary Figure S10. Mass spectra of the model peptide after residue-specific modification.** (A) Full mass spectrum of the expected Cys and Lys modified peptide ([M-H]^-^ ion) with ([M+3H]^3+^ ion) with m/z of 751.3. (B) Full mass spectrum of the Met modified peptide. The expected NTP main product ([M+3H]^3+^ ion) is with m/z of 798.2 and the OTP by-product ([M+3H]^3+^ ion) is with m/z of 756.6. (C) Full mass spectrum of the expected Asp modified peptide ([M+3H+O]^3+^ ion) with m/z of 774.9.


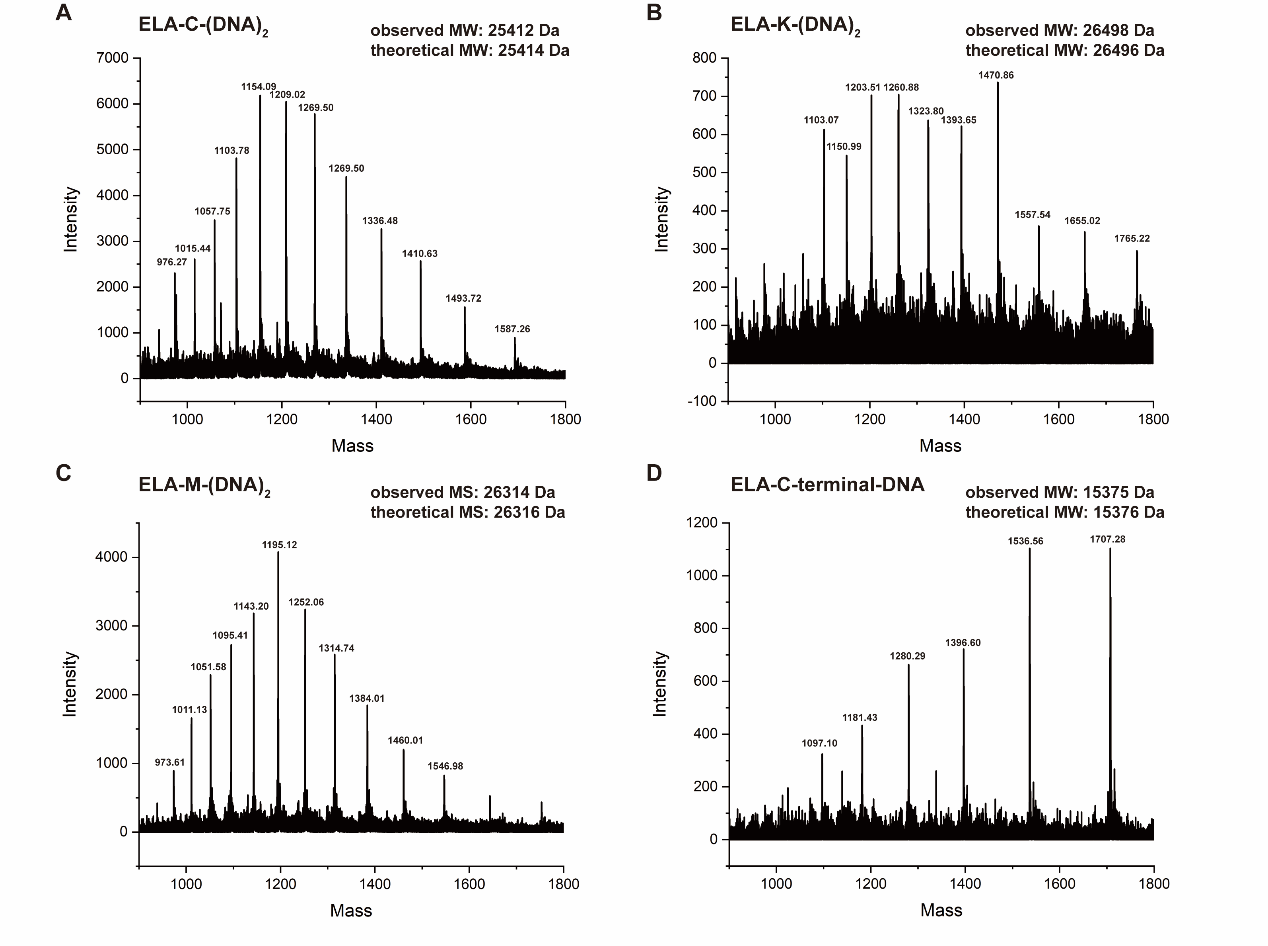


**Supplementary Figure S11. Mass spectra of the DNA barcoded ELA peptide.** (A) Full mass spectrum of ELA-C-(DNA)_2_. The theoretical MW is 25414 Da and the observed MW is 25412 Da. (B) Full mass spectrum of ELA-K-(DNA)_2_. The theoretical MW is 26496 Da and the observed MW is 26498 Da. (C) Full mass spectrum of ELA-M-(DNA)_2_. The theoretical MW is 26316 Da and the observed MW is 26314 Da. (D) Full mass spectrum of ELA-C-terminal-DNA. The theoretical MW is 15376 Da and the observed MW is 15375 Da.


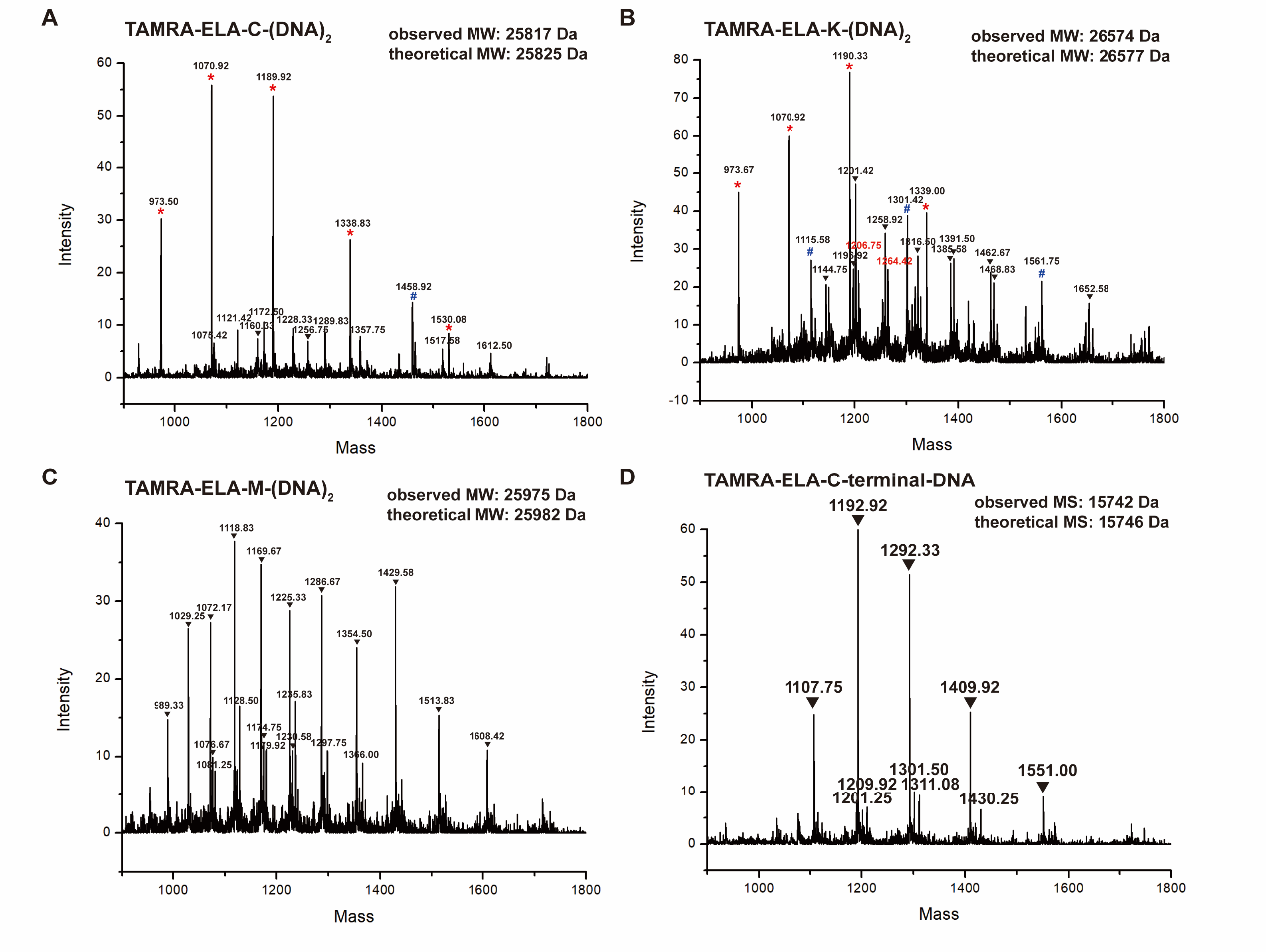


**Supplementary Figure S12. Mass spectra of the DNA barcoded TAMRA-ELA peptide.** (A) Full mass spectrum of TAMRA-ELA-C-(DNA)_2_. The asterisks labeled peaks correspond to the DNA fragment ions. The triangles labeled peaks correspond to the fragment ions releasing one Mal-DNA free radical. The pound signs labeled peak corresponds to correspond to the fragment ions releasing two Mal-DNA free radicals. The peaks without any labels are the ions of the TAMRA-ELA-C-(DNA)_2_. The theoretical MW is 25825 Da and the observed MW is 25817 Da. (B) Full mass spectrum of TAMRA-ELA-K-(DNA)_2_. The asterisks labeled peaks correspond to the DNA fragment ions. The triangles labeled peaks correspond to the fragment ions releasing one or two N-methyl maleimide free radicals. The pound signs labeled peak corresponds to the fragment ions releasing one Mal-DNA free radical. The peaks without any labels are the ions of the TAMRA-ELA-K-(DNA)_2_. The theoretical MW is 26577 Da and the observed MW is 26574 Da. (C) Full mass spectrum of TAMRA-ELA-M-(DNA)_2_. The triangles labeled peaks correspond to the fragment ions releasing one or two N-methyl maleimide free radicals. The peaks without any labels are the ions of the TAMRA-ELA-M-(DNA)_2_. The theoretical MW is 25982 Da and the observed MW is 25975 Da. (D) Full mass spectrum of TAMRA-ELA-C-teriminal-DNA. The triangles labeled peaks correspond to the fragment ions releasing two N-methyl maleimide free radicals. The peaks without any labels are the ions of the TAMRA-ELA-C-teriminal-DNA. The theoretical MW is 15746 Da and the observed MW is 15742 Da.


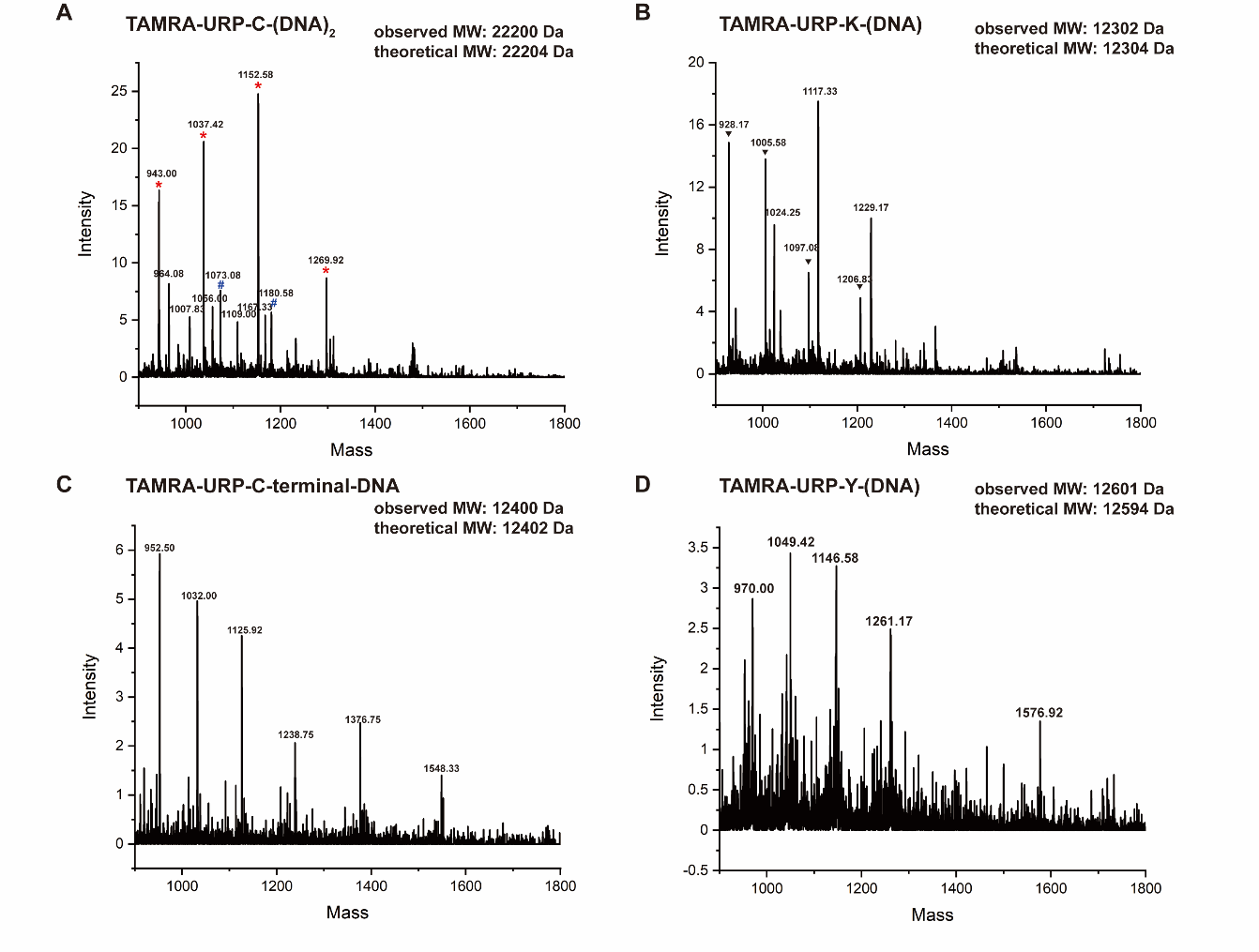


**Supplementary Figure S13. Mass spectra of the DNA barcoded TAMRA-URP.** (A) Full mass spectrum of TAMRA-URP-C-(DNA)_2_. The asterisks labeled peaks correspond to the DNA fragment ions. The pound signs labeled peaks correspond to the fragment ions releasing one Mal-DNA free radical. The peaks without any labels are the ions of the TAMRA-URP-C-(DNA)_2_. The theoretical MW is 22204 Da and the observed MW is 22200 Da. (B) Full mass spectrum of TAMRA-URP-K-(DNA). The triangles labeled peaks correspond to the fragment ions releasing two N-methyl maleimide free radicals. The peaks without any labels are the ions of the TAMRA-URP-K-(DNA). The theoretical MW is 12304 Da and the observed MW is 12302 Da. (C) Full mass spectrum of TAMRA-URP-C-teriminal-DNA. The theoretical MW is 12400 Da and the observed MW is 12402 Da. (D) Full mass spectrum of TAMRA-URP-Y-(DNA). The theoretical MW is 12594 Da and the observed MW is 12601 Da.


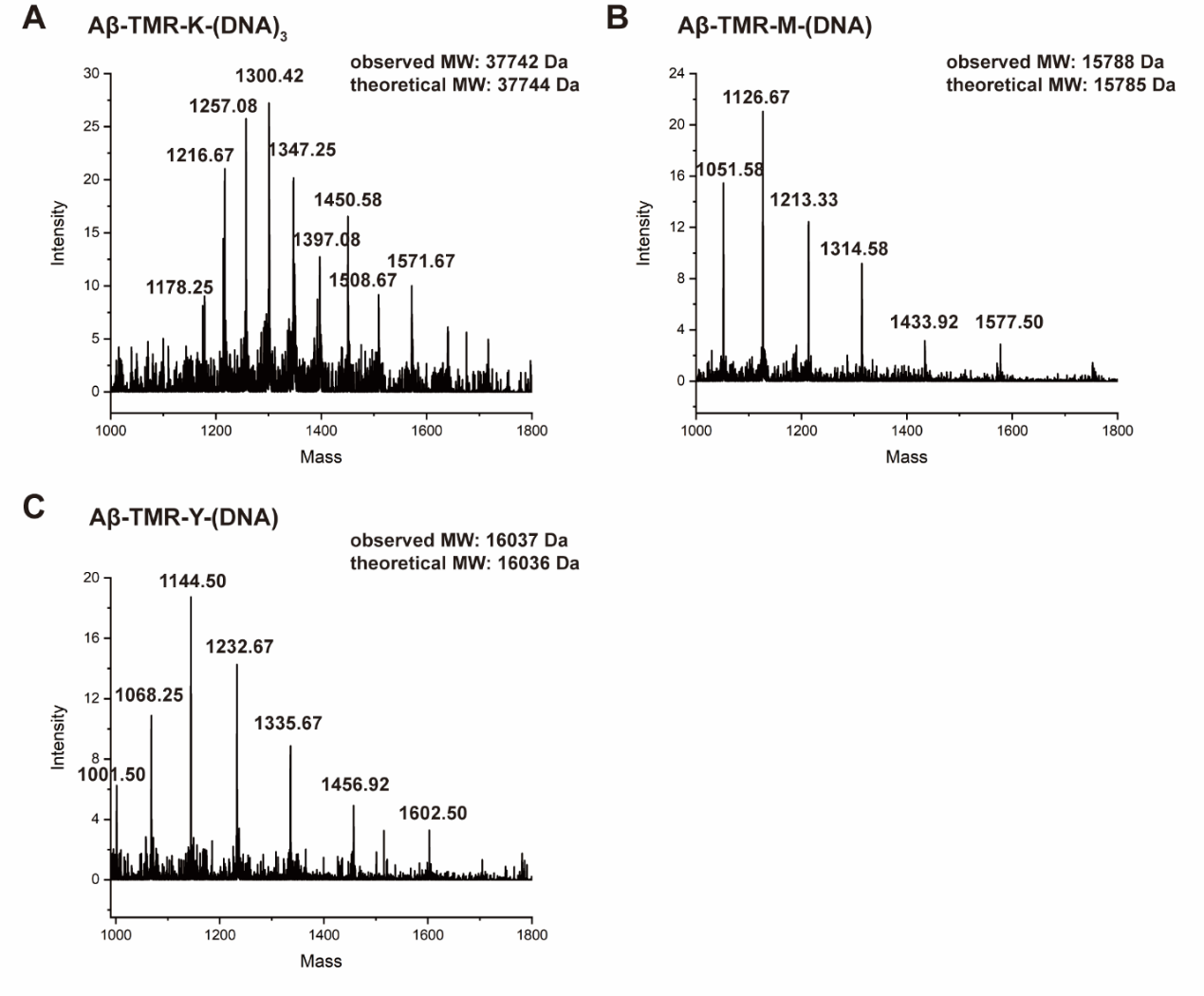


**Supplementary Figure S14. Mass spectra of the DNA barcoded Aβ-TAMRA.** (A) Full mass spectrum of Aβ-TAMRA-K-(DNA)_3_. The theoretical MW is 37744 Da and the observed MW is 37742 Da. (B) Full mass spectrum of Aβ-TAMRA-M-(DNA). The theoretical MW is 15785 Da and the observed MW is 15788 Da. (C) Full mass spectrum of Aβ-TAMRA-Y-(DNA). The theoretical MW is 16036 Da and the observed MW is 16037 Da.


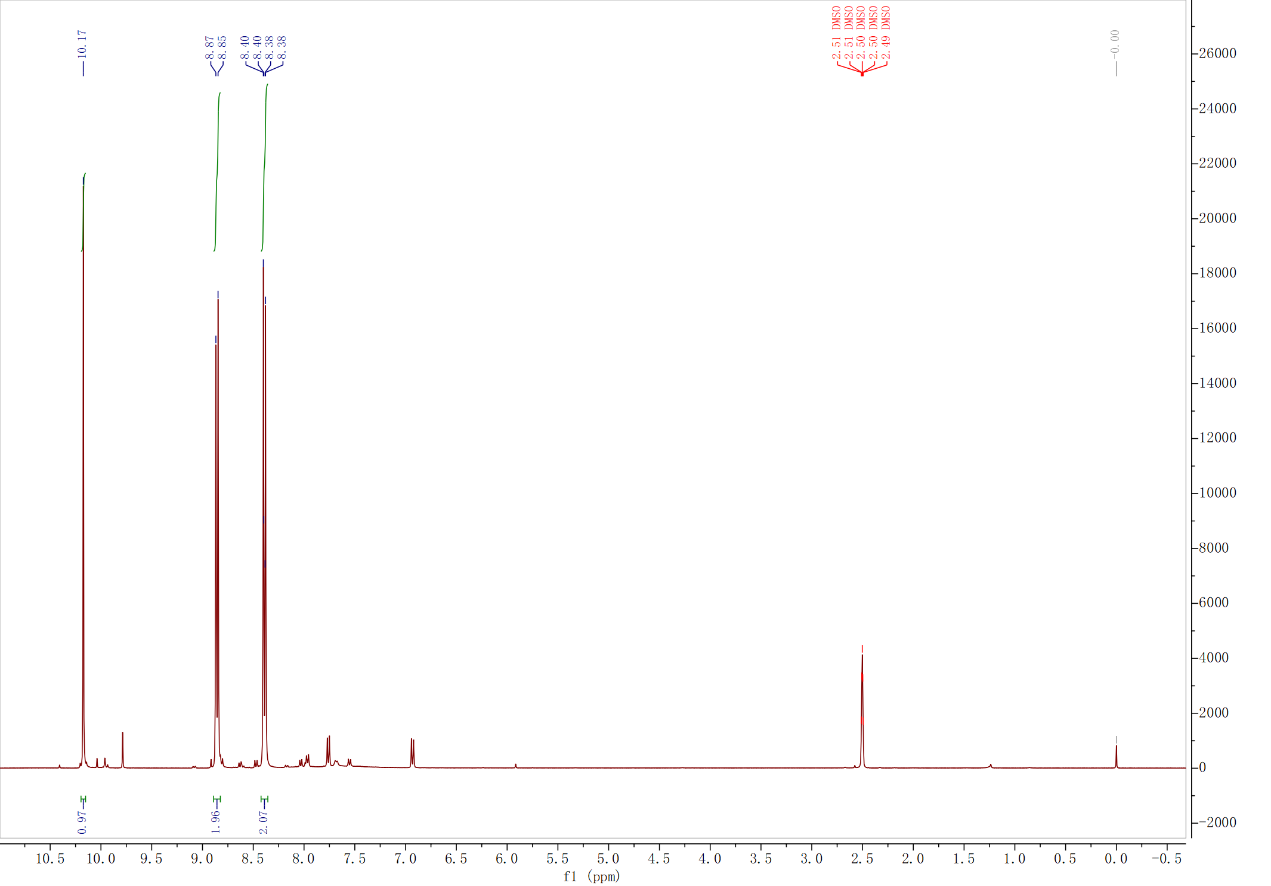


**Supplementary Figure S15. ^1^H-NMR of 4-formylbenzene diazonium hexafluorophosphate.**

**Supplementary Table S1. List of DNA oligos.**

| Name | DNA sequence (5’ to 3’) |
| --- | --- |
| 32 nt DNA template **(DNA)** | CGTGTCCTCGTTGTGAGATCACAGCATACCGT |
| Forward primer | CGTGTCCTCGTTGTG |
| Reverse primer | CGGTATGCTGTGATC |

**Supplementary Table S2. List of commercially synthetic peptides.**

| Name | Peptide sequence (N to C) | | Modification |
| --- | --- | --- | --- |
| ELA | | QRPVNLTMRRKLRKHNCLQRRCMPLHSRVPFP | No modification |
| ELA  (protected) | | QRPVNLTMRRKLRKHNCLQRRCMPLHSRVPFP | Mal-Me on Cys  Acetylation on N-terminal |
| TAMRA-ELA | | QRPVNLTMRRKLRKHNCLQRRCMPLHSRVPFP | TAMRA on N-terminal |
| URP | | ACFWKYCV | No modification |
| TAMRA-URP | | ACFWKYCV | TAMRA on N-terminal |
| Model peptide | | GAGSKGSAGSDGSCGSAGSMG | TAMRA on N-terminal  Amination on C-terminal |
| Aβ | | DAEFRHDSGYEVHHQKLVFFAEDVGSNKGAIIGLMVGGVV | No modification |
| Humanin-like 9 | | MARRGFSCLLLSTTATDLPVKRRT | No modification |
| NY-ESO-1  (157-165) | | SLLMWITQC | No modification |

**Supplementary Table S3. List of DNA barcoded peptides.**

| Name | Sequence (ignoring modifications) | Linker on peptide | Linker on DNA |
| --- | --- | --- | --- |
| ELA-C-(DNA)2 | QRPVNLTMRRKLRKHNC(DNA)LQRRC(DNA)MPLHSRVPFP | No | Mal-PEG12 |
| ELA-K-(DNA)2 | QRPVNLTMRRK(DNA)LRK(DNA)HNCLQRRCMPLHSRVPFP | PEG4-N3 | DBCO-PEG12 |
| ELA-M-(DNA)2 | QRPVNLTM(DNA)RRKLRKHNCLQRRCM(DNA)PLHSRVPFP | Ox6(N3) | DBCO-PEG12 |
| ELA-C-terminal-DNA | QRPVNLTMRRKLRKHNCLQRRCMPLHSRVPFP-DNA | PEG3-N3 | DBCO-PEG12 |
| TAMRA-ELA-C-(DNA)2 | QRPVNLTMRRKLRKHNC(DNA)LQRRC(DNA)MPLHSRVPFP | No | Mal-PEG12 |
| TAMRA-ELA-K-(DNA)2 | QRPVNLTMRRK(DNA)LRK(DNA)HNCLQRRCMPLHSRVPFP | PEG4-SAc | Mal-PEG12 |
| TAMRA-ELA-M-(DNA)2 | QRPVNLTM(DNA)RRKLRKHNCLQRRCM(DNA)PLHSRVPFP | Ox6(N3) | DBCO-PEG4 |
| TAMRA-ELA-C-terminal-DNA | QRPVNLTMRRKLRKHNCLQRRCMPLHSRVPFP-DNA | PEG3-N3 | DBCO-PEG12 |
| URP-Y-(DNA) | ACFWKY(DNA)CV | PEG4-N3 | DBCO-PEG12 |
| TAMRA-URP-C-(DNA)2 | AC(DNA)FWKYC(DNA)V | No | Mal-PEG6 |
| TAMRA-URP-K-(DNA) | ACFWK(DNA)YCV | PEG4-SAc | Mal-PEG6 |
| TAMRA-URP-C-terminal-DNA | ACFWKYCV-DNA | PEG3-N3 | DBCO-PEG4 |
| TAMRA-URP-Y-(DNA) | ACFWKY(DNA)CV | PEG4-N3 | DBCO-PEG4 |
| Aβ-TAMRA-K-(DNA)3 | DNA-DAEFRHDSGYEVHHQK(DNA)LVFFAEDVGSNK(DNA)GAIIGLMVGGVV | PEG4-SAc | Mal-PEG12 |
| Aβ-TAMRA-M-(DNA) | DAEFRHDSGYEVHHQKLVFFAEDVGSNKGAIIGLM(DNA)VGGVV | Ox6(N3) | DBCO-PEG12 |
| Aβ-TAMRA-Y-(DNA) | DAEFRHDSGY(DNA)EVHHQKLVFFAEDVGSNKGAIIGLMVGGVV | PEG4-N3 | DBCO-PEG12 |

**References**

1. Podracky CJ, An C, DeSousa A *et al.* Laboratory evolution of a sortase enzyme that modifies amyloid-β protein. *Nat Chem Biol*. 2021; **17**: 317-25.

2. Gavrilyuk J, Ban H, Nagano M *et al.* Formylbenzene diazonium hexafluorophosphate reagent for tyrosine-selective modification of proteins and the introduction of a bioorthogonal aldehyde. *Bioconjugate Chem*. 2012; **23**: 2321-28.
